# Correction: Who is engaging with lateral flow testing for COVID-19 in The UK? The COVID-19 Rapid Survey of Adherence to Interventions and Responses (CORSAIR) study

**DOI:** 10.1136/bmjopen-2021-058060corr1

**Published:** 2022-06-20

Smith LE, Potts HW, Amlôt R, *et al*. Who is engaging with lateral flow testing for COVID-19 in the UK? The COVID-19 Rapid Survey of Adherence to Interventions and Responses (CORSAIR) study. *BMJ Open* 2022;12:e058060. doi: 10.1136/bmjopen-2021-058060

This article was previously published with an error.

There is an issue with labelling for socio-economic grade. The item for this variable asks participants to state the profession of the highest earner in the household. We categorised participants into two groups: highest earner works in a manual occupation, and highest earner does not work in a manual occupation. The levels of these variables are referred to in some of our project’s manuscripts as ‘socio-economic grade C1DE’ and ‘socio-economic grade ABC1’, respectively. These would be better denoted as ‘highest earner in household works in a manual occupation’ and ‘highest earner in household does not work in a manual occupation’. This labelling error came about through multiple iterations of documents.

