# Supplementary material for: Who is engaging with lateral flow testing for COVID-19 in the UK? The COVID-19 Rapid Survey of Adherence to Interventions and Responses (CORSAIR) study
Source: BMJ Open. 2022 Feb 9;12(2):e058060. doi: 10.1136/bmjopen-2021-058060 (PMC8845094; doi:10.1136/bmjopen-2021-058060)
Supplement: Author's manuscript [file bmjopen-2021-058060.draft_revisions.pdf]

BMJ Open is committed to open peer review. As part of this commitment we make the peer review history of every article we publish publicly available.

When an article is published we post the peer reviewers' comments and the authors' responses online. We also post the versions of the paper that were used during peer review. These are the versions that the peer review comments apply to.

The versions of the paper that follow are the versions that were submitted during the peer review process. They are not the versions of record or the final published versions. They should not be cited or distributed as the published version of this manuscript.

BMJ Open is an open access journal and the full, final, typeset and author-corrected version of record of the manuscript is available on our site with no access controls, subscription charges or pay-per-view fees (http://bmjopen.bmj.com).

If you have any questions on BMJ Open's open peer review process please email info.bmjopen@bmj.com

BMJ Open

# Who is engaging with lateral flow testing for COVID-19 in the UK? The COVID-19 Rapid Survey Of Adherence To Interventions And Responses (CORSAIR) study

| | |
|---|---|
| Journal: | *BMJ Open* |
| Manuscript ID | bmjopen-2021-058060 |
| Article Type: | Original research |
| Date Submitted by the Author: | 05-Oct-2021 |
| Complete List of Authors: | Smith, Louise; Institute of Psychiatry Psychology and Neuroscience<br>Potts, Henry; University College London, Institute of Health Informatics<br>Amlot, Richard; UK Health Security Agency<br>Fear, Nicola; King's College London, Academic Centre for Defence Mental Health<br>Michie, Susan; University College London, Centre for Behaviour Change<br>Rubin, GJ; King's College London |
| Keywords: | COVID-19, Public health < INFECTIOUS DISEASES, PUBLIC HEALTH |
| | |

SCHOLARONE™
Manuscripts

*I, the Submitting Author has the right to grant and does grant on behalf of all authors of the Work (as defined in the below author licence), an exclusive licence and/or a non-exclusive licence for contributions from authors who are: i) UK Crown employees; ii) where BMJ has agreed a CC-BY licence shall apply, and/or iii) in accordance with the terms applicable for US Federal Government officers or employees acting as part of their official duties; on a worldwide, perpetual, irrevocable, royalty-free basis to BMJ Publishing Group Ltd ("BMJ") its licensees and where the relevant Journal is co-owned by BMJ to the co-owners of the Journal, to publish the Work in this journal and any other BMJ products and to exploit all rights, as set out in our licence.*

*The Submitting Author accepts and understands that any supply made under these terms is made by BMJ to the Submitting Author unless you are acting as an employee on behalf of your employer or a postgraduate student of an affiliated institution which is paying any applicable article publishing charge ("APC") for Open Access articles. Where the Submitting Author wishes to make the Work available on an Open Access basis (and intends to pay the relevant APC), the terms of reuse of such Open Access shall be governed by a Creative Commons licence – details of these licences and which Creative Commons licence will apply to this Work are set out in our licence referred to above.*

*Other than as permitted in any relevant BMJ Author's Self Archiving Policies, I confirm this Work has not been accepted for publication elsewhere, is not being considered for publication elsewhere and does not duplicate material already published. I confirm all authors consent to publication of this Work and authorise the granting of this licence.*

For peer review only

# Who is engaging with lateral flow testing for COVID-19 in the UK? The COVID-19 Rapid Survey of Adherence to Interventions and Responses (CORSAIR) study

Louise E Smith (0000-0002-1277-2564),[1,2] PhD, Henry WW Potts (0000-0002-6200-8804),[3] PhD, Richard Amlôt (0000-0003-3481-6588),[2,4] PhD, Nicola T Fear (0000-0002-5792-2925),[1,5] DPhil (Oxon), Susan Michie (0000-0003-0063-6378),[6] DPhil, G James Rubin (0000-0002-4440-0570),[1,2] PhD

1 King's College London, Institute of Psychiatry, Psychology and Neuroscience

2 NIHR Health Protection Research Unit in Emergency Preparedness and Response

3 University College London, Institute of Health Informatics

4 UK Health Security Agency, Behavioural Science and Insights Unit

5 King's Centre for Military Health Research and Academic Department of Military Mental Health

6 University College London, Centre for Behaviour Change

Corresponding author: Louise E Smith, Post-doctoral Researcher. Department of Psychological Medicine, King's College London, Weston Education Centre, Cutcombe Road, London, SE5 9RJ. Email: louise.e.smith@kcl.ac.uk. ORCID number: 0000-0002-1277-2564. Twitter handle: @louisesmith142

Henry WW Potts, Professor of Health Informatics. Institute of Health Informatics, 222 Euston Road, London, NW1 2DA.

Richard Amlôt, Head of Behavioural Science in the Behavioural Science and Insights Unit at UK Health Security Agency. Porton Down, Salisbury, Wiltshire, SP4 0JG.

Nicola T Fear, Professor of Epidemiology. Department of Psychological Medicine, King's College London, Weston Education Centre, Cutcombe Road, London, SE5 9RJ.



For peer review only - http://bmjopen.bmj.com/site/about/guidelines.xhtml

Susan Michie, Professor of Health Psychology. Centre for Behaviour Change, University College London,1-19 Torrington Place, London, WC1E 7HB.

G James Rubin, Assistant Director, NIHR Health Protection Research Unit in Emergency Preparedness and Response, King's College London, Weston Education Centre, Cutcombe Road, London, SE5 9RJ.

For peer review only



For peer review only - http://bmjopen.bmj.com/site/about/guidelines.xhtml

# ABSTRACT

Objectives: To investigate uptake of lateral flow testing, reporting of test results, and psychological, contextual, and socio-demographic factors associated with testing.

Design: Series of four fortnightly online cross-sectional surveys.

Setting: Data collected 19 April to 2 June 2021.

Participants: People who lived in England and Scotland, aged 18 years or over, excluding people who reported their most recent test was a polymerase chain reaction (PCR) test (n=6646, n≈1,600 per wave).

Main outcome measures: Having completed at least one lateral flow test (LFT) in the last seven days.

Results: We used binary logistic regressions to investigate factors associated with having taken an LFT in the last week. Increased uptake of testing was associated with being younger, female, living with a dependent child, being employed and being vaccinated. Work- and study-related factors such as having been out to work in the last week, working in a sector that adopted LFT early, and being a student were also associated with increased uptake. People who reported COVID-19 symptoms in the last week were more likely to have taken a test, as were those who had heard more about regular LFT, knew they were eligible to receive regular LFT, and agreed that LFTs were accurate and an effective way to prevent the spread of COVID-19. Factors associated with not taking a test included agreeing that you do not need to test for COVID-19 unless you have come into contact with a case, and that people who have been vaccinated do not need to be tested regularly.

Conclusions: Uptake of lateral flow testing is low. Encouraging testing through workplaces and places of study are likely to increase uptake, although care should be taken not to pressurise employees and students. Increasing knowledge that everyone is eligible for regular asymptomatic testing and addressing common misconceptions may drive uptake.


Keywords: COVID-19, antigen testing, lateral flow testing, mass asymptomatic testing, uptake, predictors

Abstract word count: 300



For peer review only - http://bmjopen.bmj.com/site/about/guidelines.xhtml

## STRENGTHS AND LIMITATIONS OF THIS STUDY

- Series of large cross-sectional nationally representative surveys investigating uptake, and factors associated with uptake, of lateral flow testing in the English population.
- One of few studies investigating psychological, contextual, and socio-demographic factors associated with uptake of testing.
- Data were collected in the weeks immediately after the recommendation that all UK adults should take two rapid, lateral flow tests per week.
- Self-reported rates of having completed a lateral flow test in the last week were higher than estimated by official agencies.
- The behaviour and beliefs of people who have signed up to complete internet surveys may not be representative of those of the general population.

For peer review only



For peer review only - http://bmjopen.bmj.com/site/about/guidelines.xhtml

# INTRODUCTION

As the UK moves to continuous management of COVID-19 instead of disaster prevention, a variety of strategies are being used to slow the spread of infection. It is thought that asymptomatic cases account for 24% of COVID-19 transmission.(1) There is limited evidence of the effectiveness of mass asymptomatic testing programmes at slowing the spread of COVID-19 as most programmes, both in the UK and abroad, have been used in conjunction with other behavioural restrictions making it impossible to quantify the impact of mass testing alone.(2, 3) However, the effectiveness of any intervention will be limited if people do not engage with that behaviour.

Since 9 April 2021, everyone in the UK has been able to access free, regular, rapid lateral flow COVID-19 testing for use when asymptomatic.(4) At the time of writing, the English and Scottish Governments are recommending twice weekly lateral flow testing for all adults. Lateral flow tests (LFTs) for asymptomatic testing can be ordered online, collected from NHS pharmacies and are supplied through schools, colleges and nurseries; some universities and other employers also offer rapid testing.(5) Results of these tests should be reported through a UK Government website.(6) Mass asymptomatic testing was piloted in Liverpool, England between 6 November 2020 and 30 April 2021. Findings indicated that 57% of residents in Liverpool took at least one rapid LFT over the course of the pilot (~6 months), but uptake was substantially lower in more deprived areas (where infection rates were higher) and among non-white minoritized ethnic groups.(7) Testing was likely driven by the intense media campaign in Liverpool, and the novelty of the testing at the time. As well as asymptomatic testing being available, all those with key COVID-19 symptoms have been eligible for a polymerase chain reaction (PCR) test since 18 May 2020.(8) There is considerable confusion among the public about the use of LFT and PCR tests when symptomatic.(9) Previous research indicates that uptake of testing when symptomatic is low.(10)

A range of factors may affect whether people engage with lateral flow testing. These can be categorised using the Capability, Opportunity, Motivation and Behaviour (COM-B) model.(11) Capability encompasses the psychological and physical capacity to engage in a behaviour. It includes, for example, knowledge as to what the appropriate behaviour is (e.g. knowing that you are eligible for regular testing) and when to enact it. Opportunity relates to factors outside the person, for example testing being required by one's employer, or



For peer review only - http://bmjopen.bmj.com/site/about/guidelines.xhtml

belonging to a group that was eligible for asymptomatic testing prior to the nationwide rollout (e.g. health and social care workers, teachers, students and people who work in transport such as hauliers) (12-15) and that, in turn, may be associated with socio-economic status or ethnicity. Motivation describes the psychological processes that energise or inhibit a behaviour and includes the perceived risk associated with a disease outbreak, which may in turn be linked to greater exposure to other people (e.g. during socialising),(16) believing that you have immunity against COVID-19,(17) believing that you could engage in a behaviour if you wanted to (self-efficacy), and perceiving the behaviour to be effective.

The aim of this study was to investigate rates of uptake of lateral flow testing and reporting of results in England and Scotland, and the psychological, contextual, and socio-demographic factors associated with testing.

## METHODS

### Design

Since January 2020, BMG Research has been conducting a series of nationally representative (UK) cross-sectional surveys (weekly or fortnightly) on behalf of the Department of Health and Social Care throughout the COVID-19 outbreak. We analysed these data as part of the CORSAIR study [the COVID-19 Rapid Survey of Adherence to Interventions and Responses study]).(10) For this study, we used data collected between 19 April and 2 June 2021 (waves 48 to 51).

### Participants

Participants (n≈2,000 per wave) were eligible for the study if they were aged 16 years or over and lived in the UK and were recruited from two specialist research panel providers, Respondi (n=50,000) and Savanta (n=31,500). Quotas were applied based on age and gender (combined) to ensure the sample was broadly representative of the population. After completing the survey, participants were then unable to participate in the subsequent three waves; thus, all participants included in this study were unique. Participants were reimbursed in points which could be redeemed in cash, gift vouchers or charitable donations (up to 70p).

We limited the sample to people living in England or Scotland as Wales and Northern Ireland are following a different testing schedule. We excluded those under 18 years of age as many would be eligible for asymptomatic testing under school testing regimes.



For peer review only - http://bmjopen.bmj.com/site/about/guidelines.xhtml

To investigate uptake of lateral flow testing, we excluded people who reported that their most recent test was a PCR test or they did not know what type their most recent test was and who reported that they had completed a PCR test or who did not know what type of test they completed after developing COVID-19 symptoms.

## Study materials

### Lateral flow testing

Participants were asked "when was the last time [they] had a test for coronavirus". Response options ranged from "within the last 24 hours" to "I've never had a coronavirus test".

People who reported having a COVID-19 test in the last week were asked a series of follow-up questions. These included how many times they had taken a COVID-19 test in the last seven days" (responses from "once" to "ten times or more"); how they received their most recent COVID-19 test (response options included receiving it from a care home, one's place of work, a school, further education college, or university, a hospital/clinical setting, having ordered it online, collecting a pack from a local test site or taking an assisted test at a local test site, or when travelling internationally); where they reported their test result, if at all (response options included the GOV.UK website, by phone with NHS Test and Trace, to one's employer, a school, further education college, or university, and to friends and family); what the result of their most recent test was (response options: "I tested positive", "I tested negative", "The test was void (inconclusive)", "I have not received my results yet"); and which type of test they had most recently taken (response options: "PCR test", "lateral flow test" or "don't know/unsure"). For this question, participants were given an explanation of each test ("PCR tests are sent to a lab for processing and results are sent to you usually by text or email. This includes home test kits which you need to mail in or drop off. Rapid lateral flow tests provide results within 30 minutes of taking the test (these might also be referred to as rapid antigen tests). Both tests involve swabbing the back of your nose and throat").

All participants were asked how much they had previously heard about "free, regular rapid testing for people even if they don't have coronavirus symptoms, which uses a technology called 'lateral flow testing'" on a four-point scale from "nothing at all" to "a great deal". They were also asked if "as far as [they knew, they were] eligible to receive rapid COVID-19 tests twice a week to check for coronavirus even if [they didn't] have symptoms (also known as lateral flow testing)?". Possible responses were "yes", "no" and "don't know".



For peer review only - http://bmjopen.bmj.com/site/about/guidelines.xhtml

### Contextual factors

All participants were asked if they had had any symptoms in the past seven days from a list of ten (new, continuous cough; high temperature / fever; runny nose; diarrhoea; nausea / feeling sick; vomiting; sneezing; loss of appetite; loss of sense of smell; loss of taste). In the final wave of data collection, this question changed to ask if participants had developed any symptoms in the past ten days (response options remained identical).

We also identified people in groups who had been eligible for asymptomatic testing before it became available to everyone (people who indicated that they worked or volunteered in health or social care, education and childcare, or transport). Participants were identified as students if they specified that their employment status was "student / on a government training programme (Nation Traineeship/Modern Apprentice)".

Participants were asked how many times in the last seven days they had been out of their home to go to work and to meet up with friends or family that they did not live with (responses capped at 30). In the last wave of data collection, this question was changed to ask how many times in the past seven days they had "left the house to go out to work (number of days)" and "met up with" friends or family they did not live with (responses capped at 30). We recoded the number of times people had been out for work into a binary variable (been out to work in the last week vs not).

### Psychological factors

Participants were asked how much, if at all, they agreed that: they were confident that lateral flow tests were accurate; regularly testing people without symptoms is an effective way to prevent COVID-19 transmission; you do not need to take a lateral flow test unless you have come into contact with a COVID-19 case; and that people who have been vaccinated do not need to be tested for COVID-19 regularly. All questions were asked on a scale from "strongly disagree" to "strongly agree".

Perceived risk of COVID-19 was measured by asking participants to what extent they thought COVID-19 posed a risk to "you personally" and "people in the UK" on a five-point scale from "no risk at all" to "major risk".

### Socio-demographic factors

Participants were asked for their sex, age, employment status, socio-economic grade, highest educational or professional qualification, ethnicity, first language and COVID-19 vaccination status. Participants were also asked whether they had any dependent children in their



For peer review only - http://bmjopen.bmj.com/site/about/guidelines.xhtml

household and whether they or a member of their household had a chronic illness. We computed a quadratic term for age. Region and index of multiple deprivation was derived from participants' postcode.(18)

Participants were also asked if they had had, or currently had, COVID-19. We recoded responses to give a binary variable (think have had COVID-19 ["I've definitely had it, and had it confirmed by a test", "I think I've probably had it"] vs think have not had COVID-19 ["I don't know whether I've had it or not", "I think I've probably not had it", "I've definitely not had it"]).

We measured financial hardship by asking participants to what extent in the past seven days they had been struggling to make ends meet, skipping meals they would usually have, and were finding their current living situation difficult (Cronbach's $\alpha$=.83).

## Ethics

This work was conducted as a service evaluation of the Department of Health and Social Care's public communications campaign and, following advice from King's College London Research Ethics Subcommittee, was exempt from ethical approval.

## Patient and public involvement

Lay members served on the advisory group for the project that developed our prototype survey material; this included three rounds of qualitative testing.(19) Due to the rapid nature of this research, the public was not involved in the further development of the materials during the COVID-19 pandemic.

## Power

A sample size of 7,000 allows a 95% confidence interval of about plus or minus 1% for the prevalence estimate for a survey item with a prevalence of 50%.

## Analysis

We report uptake of lateral flow testing, how people received their most recent test, and out-of-home behaviour following a positive test result descriptively.

We ran multivariable logistic regression analyses to investigate contextual, psychological, and socio-demographic factors associated with having completed at least one COVID-19 test in the last week, controlling for survey wave, region, gender, age (raw and quadratic), presence of a dependent child in the household, having a chronic illness oneself, having a



For peer review only - http://bmjopen.bmj.com/site/about/guidelines.xhtml

household member who has chronic illness, employment status, socio-economic grade, index of multiple deprivation, highest educational or professional qualification, ethnicity, first language, having had COVID-19 before, vaccination status, and financial hardship. For these analyses, we excluded people who reported that they did not know when their last COVID-19 test was. As we hypothesised that peoples' beliefs about the necessity for regular asymptomatic testing of people who had been vaccinated for COVID-19 might differ based on vaccination status, we investigated associations between this belief and increased uptake of testing separately in those who reported having no, one, or two doses of a COVID-19 vaccine. We also hypothesised that participants who received a positive COVID-19 test or whose test result was inconclusive may perceive the risk of COVID-19 to themselves differently to those who had received a negative test. Therefore, we also investigated associations between perceived risk to oneself and increased uptake of testing excluding people whose most recent test result was positive or inconclusive.

Due to the large number of analyses (n=16) conducted on a single outcome, we used a Bonferroni correction and only report narratively results where $p<.003$. Tables give raw *p*-values.

# RESULTS

Since the introduction of guidance recommending twice weekly asymptomatic testing for all adults, 16.9% (95% CI 16.0% to 17.8%, n=1123/6646) of people reported that they had had a lateral flow COVID-19 test in the last week, excluding those whose most recent test was a PCR test (Table 1). Of these, 65.2% had completed two or more tests in the last week (11.0% total sample).

Table 1. Uptake of lateral flow testing

| When was the last time you had a test for coronavirus? We're interested in your most recent test, even if you didn't have symptoms [Total n=6646] | % (N) | *Asked to people who reported having a covid test in the last seven days.* And how many times have you taken a test for coronavirus in the last seven days? [Total n=1123] | % (N) |
|---|---|---|---|
| Within the last 24 hours | 4.1 (273) | Once | 34.3 (385) |
| 1-3 days ago | 7.4 (492) | Twice | 47.2 (530) |
| 4-7 days ago | 5.4 (358) | Three times | 9.4 (106) |
| One to two weeks ago | 7.1 (469) | Four to five times | 3.7 (42) |
| Two to four weeks ago | 6.9 (458) | Six to seven times | 2.8 (32) |
| One month to three months ago | 10.0 (666) | Eight to nine times | 0.7 (8) |
| Three months to six months ago | 7.8 (519) | Ten times or more | 1.2 (14) |
| More than six months ago | 6.3 (417) | | |
| I've never had a coronavirus test | 43.0 (2861) | | |



For peer review only - http://bmjopen.bmj.com/site/about/guidelines.xhtml

| | | | |
|---|---|---|---|
| Don't know | 2.0 (133) | Don’t know | 0.5 (6) |

Most people reported the result of their most recent test to someone, with 64% reporting that they registered it with an official Government agency (Table 2).

Table 2. Where people register the results of their latest test

| *Asked to people who reported having a COVID-19 test in the last seven days.* How, if at all, did you report the result of your test? Tick all that apply [Total N=1123] | % (n) |
|---|---|
| I registered my result on GOV.UK | 50.0 (561) |
| I registered my result by phone with NHS Test & Trace | 17.0 (191) |
| I informed my employer | 15.4 (173) |
| I informed the school, nursery or further education college where I or a member of my family study | 6.9 (77) |
| I informed friends/family I was planning to meet after taking the test | 6.3 (71) |
| I informed friends/family I had recently met before taking the test | 5.0 (56) |
| I informed the university where I or a member of my family study | 2.5 (28) |
| Other | 2.0 (23) |
| I did not report the result to anyone | 15.8 (177) |
| Registered test result with GOV.UK or NHS Test & Trace | 64.1 (720) |
| Registered result with anyone (GOV.UK, NHS Test & Trace, one’s employer, or the school, nursery, further education college, or university where the participant or a member of their family study) | 77.2 (867) |

## Associations with increased uptake of lateral flow testing

Increased uptake of lateral flow testing was associated with: being female, younger age, having a dependent child in your household, being employed, being vaccinated, having experienced COVID-19 symptoms in the last seven to ten days, being a student, having been out to work in the last week, working in a sector that adopted lateral flow testing early (health or social care, education and childcare, travel), having heard more about regular lateral flow testing, knowing that you are eligible for regular lateral flow testing, being confident that lateral flow tests are accurate, agreeing that regularly testing people without symptoms is an effective way to prevent the spread of COVID-19, and perceiving a greater risk of COVID-19 to people in the UK (Table 4).

Not having had a test was associated with not knowing that you were eligible for regular lateral flow testing, agreeing that you only need to take a lateral flow test if you have come into contact with somebody who has COVID-19, and that people who have been vaccinated do not need to be tested for coronavirus regularly (in people who reported at least one dose of the vaccine; Table 4). There was significant variation by region, with Scotland showing lower uptake of lateral flow testing.



For peer review only - http://bmjopen.bmj.com/site/about/guidelines.xhtml

Table 4. Factors associated with having completed at least one COVID-19 test in the last week. Bolding indicates findings significant at p<0.003.

| Factor | Level | Had not completed a COVID-19 test in the last week, n (%) [total n=5390] | Had completed a lateral flow COVID-19 test in the last week, n (%) [total n=1123] | Adjusted odds ratio (95% CI) for having completed a LFT † | p-value |
|---|---|---|---|---|---|
| Survey wave | 19 to 21 April 2021 (wave 48) | 1381 (84.6) | 252 (15.4) | Reference | - |
| | 4 to 5 May 2021 (wave 49) | 1334 (81.3) | 306 (18.7) | 1.13 (0.93 to 1.38) | .22 |
| | 17 to 19 May 2021 (wave 50) | 1326 (82.6) | 280 (17.4) | 1.04 (0.85 to 1.27) | .69 |
| | 1 to 2 June 2021 (wave 51) | 1349 (82.6) | 285 (17.4) | 0.97 (0.79 to 1.19) | .78 |
| | Overall | - | - | $\chi^2(3)$=2.8 | .42 |
| Region | East Midlands | 443 (83.6) | 87 (16.4) | Reference | - |
| | East of England | 564 (80.3) | 138 (19.7) | 1.23 (0.90 to 1.68) | .19 |
| | London | 638 (80.7) | 153 (19.3) | 1.13 (0.83 to 1.55) | .43 |
| | North East | 251 (81.8) | 56 (18.2) | 1.22 (0.83 to 1.80) | .31 |
| | North West | 669 (84.2) | 126 (15.8) | 1.02 (0.75 to 1.40) | .89 |
| | Scotland | 608 (90.3) | 65 (9.7) | **0.53 (0.37 to 0.76)** | **.001** |
| | South East | 726 (80.3) | 178 (19.7) | 1.24 (0.92 to 1.67) | .15 |
| | South West | 504 (84.3) | 94 (15.7) | 1.04 (0.74 to 1.44) | .83 |
| | West Midlands | 489 (79.5) | 126 (20.5) | 1.35 (0.98 to 1.85) | .07 |
| | Yorkshire and the Humber | 498 (83.3) | 100 (16.7) | 1.08 (0.78 to 1.50) | .65 |
| | Overall | - | - | **$\chi^2(9)$=35.6** | **<.001** |
| Gender | Male | 2508 (85.0) | 442 (15.0) | Reference | - |
| | Female | 2868 (80.9) | 676 (19.1) | 1.32 (1.14 to 1.51) | **<.001** |
| Age | Raw age | N=5390, M=50.6, SD=16.5 | N=1123, M=44.9, SD=15.9 | 0.76 (0.72 to 0.81) | **<.001** |
| Age – quadratic (age-mean)$^2$ | - | - | - | 1.0003 (1.0000 to 1.0006) | .06 |
| Dependent child in household | None | 3829 (85.3) | 660 (14.7) | Reference | - |
| | Child present | 1561 (77.1) | 463 (22.9) | **1.29 (1.10 to 1.51)** | **.001** |
| Has a chronic illness (oneself) | None | 3974 (82.2) | 819 (17.8) | Reference | - |
| | Present | 1478 (83.6) | 209 (16.4) | 1.18 (1.00 to 1.39) | .05 |
| Household member has chronic illness | None | 4482 (82.7) | 936 (17.3) | Reference | - |
| | Present | 790 (82.0) | 173 (18.0) | 1.04 (0.86 to 1.26) | .71 |
| Employment status | Not working | 2544 (88.5) | 330 (11.5) | Reference | - |
| | Working | 2783 (78.1) | 781 (21.9) | **1.94 (1.63 to 2.32)** | **<.001** |
| Socio-economic grade | ABC1 | 3844 (83.6) | 754 (16.4) | Reference | - |
| | C2DE | 1438 (80.5) | 348 (19.5) | 1.10 (0.94 to 1.29) | .21 |

For peer review only



For peer review only - http://bmjopen.bmj.com/site/about/guidelines.xhtml

| | | | | | |
|---|---|---|---|---|---|
| Index of multiple deprivation | 1st quartile (least deprived) to 4th quartile (most deprived) | N=5390, M=2.6, SD=1.1 | N=1123, M=2.6, SD=1.1 | 0.93 (0.87 to 0.99) | .02 |
| Highest educational or professional qualification | GCSE/vocational/A-level/No formal qualifications | 3625 (82.9) | 749 (17.1) | Reference | - |
| | Degree or higher (Bachelors, Masters, PhD) | 1765 (82.5) | 374 (17.5) | 0.91 (0.78 to 1.07) | .24 |
| Ethnicity | White British | 4549 (82.7) | 954 (17.3) | Reference | - |
| | White other | 323 (83.9) | 62 (16.1) | 0.87 (0.61 to 1.25) | .45 |
| | Black and minority ethnicity | 484 (82.2) | 105 (17.8) | 0.83 (0.64 to 1.08) | .16 |
| | Overall | - | - | $\chi^2(2)$=2.1 | .34 |
| English as first language | No | 415 (83.2) | 84 (16.8) | Reference | - |
| | Yes | 4975 (82.7) | 1039 (17.3) | 1.19 (0.86 to 1.65) | .29 |
| Had COVID-19 before | Think not | 4600 (83.8) | 888 (16.2) | Reference | - |
| | Think yes | 790 (77.1) | 235 (22.9) | 1.23 (1.03 to 1.47) | .02 |
| Vaccination status | Not vaccinated | 1628 (82.9) | 336 (17.1) | Reference | - |
| | 1 dose | 1937 (83.2) | 392 (16.8) | **1.52 (1.25 to 1.86)** | **<.001** |
| | 2 doses | 1825 (82.2) | 395 (17.8) | **2.45 (1.96 to 3.07)** | **<.001** |
| | Overall | - | - | **$\chi^2(2)$=61.7** | **<.001** |
| Financial hardship | Range 3 (least) to 15 (most) | N=5311, M=7.3, SD=3.0 | N=1107, M=7.5, SD=3.0 | 0.99 (0.97 to 1.01) | .38 |
| COVID-19 symptoms in last week / ten days | No | 5258 (83.2) | 1061 (16.8) | Reference | - |
| | Yes | 132 (68.0) | 62 (32.0) | **1.89 (1.34 to 2.66)** | **<.001** |
| Being a student | No | 5189 (83.0) | 1061 (17.0) | Reference | - |
| | Yes | 138 (73.4) | 50 (26.6) | **2.65 (1.76 to 4.00)** | **<.001** |
| Been out to work in last week | No | 3702 (88.3) | 490 (11.7) | Reference | - |
| | Yes | 1688 (72.7) | 633 (27.3) | **2.30 (1.94 to 2.73)** | **<.001** |
| Number of times been out to meet people from another household socially | Range 0 to 30 | N=5390, M=0.9, SD=1.5, median=0 | N=1123, M=1.2, SD=1.6, median=1 | 1.05 (1.01 to 1.10) | .03 |
| Work in a sector that adopted LFT early | No | 4700 (86.3) | 744 (13.7) | Reference | - |
| | Yes | 690 (64.5) | 379 (35.5) | **2.54 (2.14 to 3.02)** | **<.001** |
| Amount heard about regular LFT | 4-point scale from “nothing at all” to “a great deal” | N=5253, M=2.8, SD=0.8 | N=1112, M=3.3, SD=0.7 | **2.28 (2.06 to 2.51)** | **<.001** |
| As far as you know, are you eligible to receive rapid COVID-19 tests twice a week to check for coronavirus even if you don’t have symptoms (also known as lateral flow testing)? | No | 928 (90.5) | 97 (9.5) | Reference | - |
| | Don’t know | 1718 (95.0) | 91 (5.0) | **0.59 (0.43 to 0.8)** | **.001** |
| | Yes | 2744 (74.6) | 935 (25.4) | **2.98 (2.35 to 3.78)** | **<.001** |
| | Overall | - | - | **$\chi^2(2)$=240.5** | **<.001** |
| I am confident that lateral flow tests are accurate | 5-point scale from “strongly disagree” to “strongly agree” | N=5131, M=3.3, SD=1.0 | N=1097, M=3.6, SD=0.9 | **1.40 (1.29 to 1.51)** | **<.001** |
| Regularly testing people without symptoms is an effective way to prevent the spread of coronavirus | 5-point scale from “strongly disagree” to “strongly agree” | N=5225, M=3.9, SD=0.9 | N=1115, M=4.3, SD=0.8 | **1.96 (1.77 to 2.16)** | **<.001** |



For peer review only - http://bmjopen.bmj.com/site/about/guidelines.xhtml

| | | | | | |
|---|---|---|---|---|---|
| I do not need to take a lateral flow test unless I have come into contact with somebody who has coronavirus | 5-point scale from "strongly disagree" to "strongly agree" | N=5061, M=2.6, SD=1.0 | N=1114, M=2.0, SD=1.1 | **0.51 (0.47 to 0.55)** | **<.001** |
| People who have been vaccinated do not need to be tested for coronavirus regularly | 5-point scale from "strongly disagree" to "strongly agree" | | | | |
| | In people who have not been vaccinated | N=1480, N=2.8, SD=1.1 | N=329, M=2.7, SD=1.1 | 0.90 (0.80 to 1.01) | .08 |
| | In people who have had one vaccine dose | N=1790, M=2.5, SD=1.0 | N=385, M=2.0, SD=1.1 | **0.54 (0.47 to 0.61)** | **<.001** |
| | In people who have had two vaccine doses | N=1644, M=2.6, SD=1.0 | N=392, M=2.0, SD=1.1 | **0.53 (0.47 to 0.60)** | **<.001** |
| Perceived risk of COVID-19 to self | 5-point scale from "no risk at all" to "major risk" | N=5343, M=3.0, SD=1.1 | N=1117, M=3.0, SD=1.1 | 1.04 (0.98 to 1.11) | .23 |
| | Excluding people who tested positive and whose test result was inconclusive | N=5343, M=3.0, SD=1.1 | N=1058, M=3.0, SD=1.1 | 1.03 (0.96 to 1.10) | .42 |
| Perceived risk of COVID-19 to people in the UK | 5-point scale from "no risk at all" to "major risk" | N=5326, M=3.5, SD=1.0 | N=1114, M=3.6, SD=0.9 | **1.13 (1.05 to 1.22)** | **.001** |

† Adjusting for wave, region, gender, age (raw and quadratic), presence of a dependent child in the household, having a chronic illness oneself, having a household member who has chronic illness, employment status, socio-economic grade, index of multiple deprivation, highest educational or professional qualification, ethnicity, first language, having had COVID-19 before, vaccination status, and financial hardship.

Abbreviations: LFT=lateral flow test



For peer review only

# DISCUSSION

These data suggest that uptake of lateral flow testing is low, with approximately 17% of the sample reporting having a test in the last week. This is slightly lower than another survey finding that 25% of English and Scottish adults reported taking regular COVID-19 tests (defined as at least once or twice a week; data collected 29 July 2021) although that was not in a nationally representative sample.(20) These data are not directly comparable with the Liverpool pilot, which reported uptake of testing over the complete duration of the pilot (almost 6 months), rather than uptake of people per week.(7) In the first month of the pilot, 35% of people reported having taken up either LFT or PCR test.(21) Analyses of tests reported to the UK Government indicate that the number of LFTs registered had steadily declined from approximately 5.7 million LFTs (15 to 21 April 2021 (22)) to around 3.5 million (27 May and 2 June 2021).(23) This number includes tests taken by children and does not include tests that have not been officially registered on the UK Government website. However, our data would imply around 10 million LFTs should be reported each week by people aged over 17 years in England and Scotland alone, suggesting that our survey respondents may be more compliant (uptake of testing and / or reporting of testing) than the general population. This is corroborated by official figures estimating that approximately 21% of lateral flow tests are reported.(24) Our data indicate that 64% of participants' most recent tests had been registered with an official Government agency.

Factors related to employment or study were associated with uptake of lateral flow testing, with people being more likely to report having a test in the last week if they were employed, had been out to work in the last week, if they worked in a sector that recommended asymptomatic testing before the national guidance was implemented, and if they were a student. This could be because people were encouraged or compelled to take tests through their workplace, because they were more familiar with testing, or because they were more worried or perceived a greater risk of exposure to COVID-19 as they were going out to their place of work or study.(25) The current findings suggest that encouraging employees to take tests could drive uptake. However, this should be approached with caution. There are ethical issues to consider in employers putting pressure on their employees, and there is a potential resulting lack of income if workers are unable to attend their place of work if they decline.(26) Mandation of testing may result in negative attitudes towards testing becoming more entrenched.(27) Uptake could also be increased by making testing easier, e.g. at or very



For peer review only - http://bmjopen.bmj.com/site/about/guidelines.xhtml

near to places of work or study, drop-in rather than appointment-based and with explicit paid time off for testing.

Uptake of lateral flow testing was higher in people who reported experiencing COVID-19-like symptoms in the last week. People with COVID-19-like symptoms should request a PCR, rather than rely on an LFT. It is clear that this requirement is not always being followed.(9) Communications should emphasise that people with key COVID-19 symptoms should request a PCR test, as should those who test positive using an LFT.

Socio-demographic factors associated with uptake of lateral flow testing included being younger, living with a dependent child, lower socio-economic grade, and thinking that you have had COVID-19 before. Previous research has found these factors to be consistently associated with non-adherence to behaviours that prevent the spread of COVID-19.(10, 17, 28) However, the association between increased lateral flow testing and lower age has also been found in other data.(20) One possible explanation may be that younger people are less likely to work from home.(26, 29) Therefore, these findings may be an artefact of people testing in relation to their work or study. Official figures of registered tests indicate that asymptomatic testing in school aged children, who are "expected to test twice weekly" (15) under the supervision of their parents, is driving uptake, with numbers of tests conducted falling during the school holidays.(23) Parents may be likely to test themselves for COVID-19 while supervising their child's test. We are unsure why those who think they have had COVID-19 before are more likely to engage with lateral flow testing. Increased uptake of testing was associated with having been vaccinated. This may reflect general adherence, with those being more likely to engage in preventive behaviours also being more likely to be vaccinated (itself a preventive behaviour).

These data indicate that people were more likely to engage in lateral flow testing if they had heard more about, and knew they were eligible for, regular LFTs. This is consistent with uptake of preventive behaviours in previous pandemics.(30) Similar to predictions from the Protection Motivation Theory,(31) perceiving testing to be more accurate and effective were associated with increased uptake.(32) Conversely, people who agreed that you only need to take a test if you have come into contact with a COVID-19 case, and that people who have been vaccinated do not need to be tested regularly were less likely to have taken a test in the last week. The latter belief was particularly strongly associated with low uptake in those who had been vaccinated. Taken together, these results suggest that media campaigns raising



For peer review only - http://bmjopen.bmj.com/site/about/guidelines.xhtml

awareness that all adults are eligible for the mass asymptomatic testing programme are likely to increase uptake.

Although socio-demographic characteristics of the sample were broadly reflective of the UK population, we cannot be certain that the behaviour and beliefs of those that complete internet surveys are representative of those of the general population. This is reflected in the higher reporting of lateral flow tests in our sample compared to that reported by official agencies. However, associations within the data are still likely to be informative.(33) Since data reflected self-reported behaviour, reports may be biased, and influenced by social desirability or poor recall. Given that we asked about behaviour in the past week, the influence of poor recall should be low. We also mitigated this by defining uptake as having completed one test in the last week, while Government guidelines suggest two COVID-19 tests per week should be completed. Although we have data on where participants received their lateral flow tests, we did not ask why they took their most recent tests.

Our study suggests that uptake of lateral flow testing in the population is low. People with symptoms were more likely to have completed a test in the past week. Communications should highlight that people with COVID-19 symptoms should request a PCR test rather than taking an LFT. Work- and study-related factors are associated with uptake of lateral flow testing. Encouragement of employees and students, especially those attending their place of work or study, to engage in asymptomatic testing may increase uptake. However, employers and educational institutions should exercise caution so as not to place undue pressure on employees and students to test. Interventions to prevent the spread of COVID-19 are unlikely to be effective if people do not engage with the behaviour. Ensuring that people know they are eligible for regular asymptomatic testing may drive uptake. These results suggest that communications emphasising that lateral flow testing is important regardless of whether you think you have come into contact with somebody who has COVID-19, and that people who have been vaccinated still need to be testing regularly for COVID-19 may also increase uptake.

For peer review only



For peer review only - http://bmjopen.bmj.com/site/about/guidelines.xhtml

## CONTRIBUTORSHIP STATEMENT

All authors conceptualised the study and contributed to survey materials. LS completed analyses with guidance from HWWP and GJR. LS wrote the first draft of the manuscript. All authors contributed to, and approved, the final manuscript. GJR is guarantor. The corresponding author attests that all listed authors meet authorship criteria and that no others meeting the criteria have been omitted.


## FUNDING SOURCES

This work was funded by the National Institute for Health Research (NIHR) Health Services and Delivery Research programme. Surveys were commissioned and funded by Department of Health and Social Care (DHSC), with the authors providing advice on the question design and selection. LS, RA and GJR are supported by the National Institute for Health Research Health Protection Research Unit (NIHR HPRU) in Emergency Preparedness and Response, a partnership between the UK Health Security Agency, King's College London and the University of East Anglia. RA is also supported by the NIHR HPRU in Behavioural Science and Evaluation, a partnership between the UK Health Security Agency and the University of Bristol. HWWP receives funding from Public Health England and has from NHS England. NTF is part funded by a grant from the UK Ministry of Defence. The views expressed are those of the authors and not necessarily those of the NIHR, UK Health Security Agency, the Department of Health and Social Care or the Ministry of Defence. The Department of Health and Social Care funded data collection.


## COMPETING INTERESTS DECLARATION

All authors have completed the ICMJE uniform disclosure form at www.icmje.org/coi_disclosure.pdf and declare: all authors had financial support from NIHR for the submitted work; RA is an employee of the UK Health Security Agency; HWWP receives additional salary support from Public Health England and NHS England; HWWP receives consultancy fees to his employer from Ipsos MORI and has a PhD student who works at and has fees paid by Astra Zeneca; no other financial relationships with any organisations that might have an interest in the submitted work in the previous three years; no other relationships or activities that could appear to have influenced the submitted work. NTF is a participant of an independent group advising NHS Digital on the release of patient data.



For peer review only - http://bmjopen.bmj.com/site/about/guidelines.xhtml

For peer review only

All authors are participants of the UK's Scientific Advisory Group for Emergencies or its subgroups.

## ETHICAL APPROVAL

This work was conducted as part of service evaluation of the marketing and communications run by the Department of Health and Social Care, and so did not require ethical approval.

## DATA SHARING STATEMENT

No additional data are available from the authors.

## TRANSPARENCY DECLARATION

The authors affirm that the manuscript is an honest, accurate, and transparent account of the study being reported; that no important aspects of the study have been omitted; and that any discrepancies from the study as originally planned have been explained.

Surveys were commissioned and funded by DHSC, with the authors providing advice on the question design and selection. DHSC had no role in analysis, decision to publish, or preparation of the manuscript. Preliminary results were made available to DHSC.

## DISSEMINATION DECLARATION

Dissemination of survey results to participants is not possible due to the anonymous nature of data collection.

## LICENSE

A CC BY license is required. The Corresponding Author has the right to grant on behalf of all authors and does grant on behalf of all authors, a worldwide licence (the BMJ license) to the Publishers and its licensees in perpetuity, in all forms, formats and media (whether known now or created in the future), to i) publish, reproduce, distribute, display and store the Contribution, ii) translate the Contribution into other languages, create adaptations, reprints, include within collections and create summaries, extracts and/or, abstracts of the Contribution and convert or allow conversion into any format including without limitation audio, iii) create any other derivative work(s) based in whole or part on the on the Contribution, iv) to exploit all subsidiary rights to exploit all subsidiary rights that currently exist or as may exist in the future in the Contribution, v) the inclusion of electronic links from the Contribution to third



For peer review only - http://bmjopen.bmj.com/site/about/guidelines.xhtml

party material where-ever it may be located; and, vi) licence any third party to do any or all of the above. All research articles will be made available on an open access basis (with authors being asked to pay an open access fee—see copyright, open access, and permission to reuse). The terms of such open access shall be governed by a Creative Commons licence—details as to which Creative Commons licence will apply to the research article are set out in our worldwide licence referred to above.

For peer review only



For peer review only - http://bmjopen.bmj.com/site/about/guidelines.xhtml

# REFERENCES


1. Johansson MA, Quandelacy TM, Kada S, Prasad PV, Steele M, Brooks JT, et al. SARS-CoV-2 Transmission From People Without COVID-19 Symptoms. *JAMA Netw Open*. 2021;4(1):e2035057.

2. Wise J. Covid-19: Concerns persist about purpose, ethics, and effect of rapid testing in Liverpool. *BMJ*. 2020;371:m4690.

3. Pavelka M, Van-Zandvoort K, Abbott S, Sherratt K, Majdan M, group CC-w, et al. The impact of population-wide rapid antigen testing on SARS-CoV-2 prevalence in Slovakia. *medRxiv*. 2021:2020.12.02.20240648.

4. Department of Health and Social Care. New campaign urges public to get tested twice a week. 2021. Available from: https://www.gov.uk/government/news/new-campaign-urges-public-to-get-tested-twice-a-week.

5. NHS. Regular rapid lateral flow coronavirus (COVID-19) tests. 2021. Available from: https://www.nhs.uk/conditions/coronavirus-covid-19/testing/regular-rapid-coronavirus-tests-if-you-do-not-have-symptoms/.

6. GOV.UK. Report a COVID-19 rapid lateral flow test result. 2021.Available from: https://www.gov.uk/report-covid19-result.

7. University of Liverpool, NHS Test and Trace, Joint Biosecurity Centre, Public Health England, Statistics OfN. Liverpool Covid-SMART Community Testing Pilot; Evaluation Report. University of Liverpool. 2021. Available from: https://www.liverpool.ac.uk/media/livacuk/research/Mass,testing,evaluation.pdf.

8. Everyone in the United Kingdom with symptoms now eligible for coronavirus tests [press release]. 2020. Available from: https://www.gov.uk/government/news/everyone-in-the-united-kingdom-with-symptoms-now-eligible-for-coronavirus-tests.

9. Smith LE, Potts HWW, Amlôt R, Fear NT, Michie S, Rubin GJ. Do members of the public think they should use lateral flow tests or PCR tests when they have COVID-19-like symptoms? The COVID-19 Rapid Survey of Adherence to Interventions and Responses [CORSAIR] study. *Public Health*. 2021;198:260-262.




For peer review only - http://bmjopen.bmj.com/site/about/guidelines.xhtml

10. Smith LE, Potts HWW, Amlôt R, Fear NT, Michie S, Rubin GJ. Adherence to the test, trace, and isolate system in the UK: results from 37 nationally representative surveys. *BMJ*. 2021;372:n608.

11. Michie S, van Stralen MM, West R. The behaviour change wheel: a new method for characterising and designing behaviour change interventions. *Implement Sci*. 2011;6:42.

12. Chris Whitty, Stephen Powis. Asymptomatic NHS Staff Testing. 2020. Available from: https://cdn.ps.emap.com/wp-content/uploads/sites/3/2020/11/Prof-Chris-Whitty-Prof-Stephen-Powis-to-Chair-Asymptomatic-testing-of-NHS-Staff-09.11.20.pdf-FOR-WEBSITE.pdf.

13. Department for Transport. Get a coronavirus (COVID-19) test if you're an HGV or van driver. 2021. Available from: https://www.gov.uk/guidance/get-a-coronavirus-covid-19-test-if-youre-an-hgv-or-van-driver#history.

14. NHS Test and Trace. COVID-19 National Testing Programme: Schools & Colleges handbook. 2020. Available from: https://schoolsweek.co.uk/wp-content/uploads/2020/12/Schools_Colleges_Testing-Handbook_version-3.3-Copy.pdf.

15. Department for Education. Coronavirus (COVID-19) asymptomatic testing in schools and colleges. 2021. Available from: https://www.gov.uk/government/publications/coronavirus-covid-19-asymptomatic-testing-in-schools-and-colleges/coronavirus-covid-19-asymptomatic-testing-in-schools-and-colleges.

16. Dryhurst S, Schneider CR, Kerr J, Freeman ALJ, Recchia G, van der Bles AM, et al. Risk perceptions of COVID-19 around the world. *J Risk Res*. 2020:1-13.

17. Smith LE, Mottershaw AL, Egan M, Waller J, Marteau TM, Rubin GJ. The impact of believing you have had COVID-19 on self-reported behaviour: Cross-sectional survey. *PLoS One*. 2020;15(11):e0240399.

18. Ministry of Housing Communities and Local Government. The English Indices of Deprivation 2019 (IoD2019). 2019. Available from: https://assets.publishing.service.gov.uk/government/uploads/system/uploads/attachment_data/file/835115/IoD2019_Statistical_Release.pdf.

19. Rubin GJ, Bakhshi S, Amlot R, Fear N, Potts HWW, Michie S. The design of a survey questionnaire to measure perceptions and behaviour during an influenza pandemic:

For peer review only

the Flu TElephone Survey Template (FluTEST). *Health Services and Delivery Research*. 2014;2(41).

20. YouGov. At the current time, are you or are you not taking regular Covid-19 tests (at least once or twice a week)? 2021. Available from: https://yougov.co.uk/topics/health/survey-results/daily/2021/07/29/f6d26/2.

21. Department of Health and Social Care, NHS Test and Trace, British Army, Public Health england, Liverpool City Council, University of Liverpool, et al. Liverpool Covid-SMART (Systematic Meaningful Asymptomatic Repeated Testing) Pilot; Addressing SARS-CoV-2 transmission and harms from Covid-19 restrictions, as one system. 2020 Available from: https://assets.publishing.service.gov.uk/government/uploads/system/uploads/attachment_data/file/950695/s0958-liverpool-covid-smart-evaluation.pdf.

22. Department of Health and Social Care. Weekly statistics for NHS Test and Trace (England) and coronavirus testing (UK): 15 April to 21 April 2021. 2021. Available from: https://assets.publishing.service.gov.uk/government/uploads/system/uploads/attachment_data/file/982248/Test_and_Trace_Week47.pdf.

23. Department of Health and Social Care. Weekly statistics for rapid asymptomatic testing in England: 27 May to 2 June 2021. 2021. Available from: https://www.gov.uk/government/publications/weekly-statistics-for-nhs-test-and-trace-england-27-may-to-2-june-2021/weekly-statistics-for-rapid-asymptomatic-testing-in-england-27-may-to-2-june-2021#lfd-tests-conducted-england1.

24. Department of Health and Social Care. Management information on lateral flow device (LFD) test registration rates: 22 July 2021. 2021. Available from: https://www.gov.uk/government/publications/management-information-on-lfd-test-registration-rates-22-july-2021/management-information-on-lateral-flow-device-lfd-test-registration-rates-22-july-2021#estimated-proportion-of-lfd-tests-dispatched-which-have-been-registered.

25. Kim Parker, Juliana Menasce Horowitz, Minkin R. How the Coronavirus Outbreak Has – and Hasn't – Changed the Way Americans Work. 2020. Available from: https://www.pewresearch.org/social-trends/2020/12/09/how-the-coronavirus-outbreak-has-and-hasnt-changed-the-way-americans-work/.



For peer review only - http://bmjopen.bmj.com/site/about/guidelines.xhtml

26. Michie S, Potts HWW, West R, Amlôt R, Smith LE, Fear NT, et al. Factors associated with nonessential workplace attendance during the Covid-19 pandemic in the UK in early 2021: evidence from cross-sectional surveys. *Public Health*. 2021;198:106-113.

27. Smith LE, Hodson A, Rubin GJ. Parental attitudes towards mandatory vaccination; a systematic review. *Vaccine*. 2021;39(30):4046-53.

28. Wright L, Steptoe A, Fancourt D. Patterns of compliance with COVID-19 preventive behaviours: a latent class analysis of 20,000 UK adults. *medRxiv*. 2021:2021.03.16.21253717.

29. Office for National Statistics. Coronavirus and homeworking in the UK: April 2020. 2020. Available from: https://www.ons.gov.uk/employmentandlabourmarket/peopleinwork/employmentandemployeetypes/bulletins/coronavirusandhomeworkingintheuk/april2020#homeworking-by-occupation.

30. Rubin GJ, Potts HWW, Michie S. The impact of communications about swine flu (influenza A H1N1v) on public responses to the outbreak: results from 36 national telephone surveys in the UK. *Health Technol Asses*. 2010;14(34):183-266.

31. Rogers RW, Prentice-Dunn S. Protection motivation theory. In: Gochman DS, editor. Handbook of health behavior research 1: Personal and social determinants: Plenum Press; 1997.

32. van Loenhout JAF, Vanderplanken K, Scheen B, Van den Broucke S, Aujoulat I. Determinants of adherence to COVID-19 measures among the Belgian population: an application of the protection motivation theory. *Archives of Public Health*. 2021;79(1):74.

33. Kohler U. Possible Uses of Nonprobability Sampling for the Social Sciences. *Survey Methods: Insights from the Field*. 2019. Available from: https://surveyinsights.org/?p=10981.

For peer review only



For peer review only - http://bmjopen.bmj.com/site/about/guidelines.xhtml

STROBE Statement—checklist of items that should be included in reports of observational studies

| | Item No | Recommendation | Page No |
|---|---|---|---|
| **Title and abstract** | 1 | (*a*) Indicate the study's design with a commonly used term in the title or the abstract | 1, 4 |
| | | (*b*) Provide in the abstract an informative and balanced summary of what was done and what was found | 4 |
| **Introduction** | | | |
| Background/rationale | 2 | Explain the scientific background and rationale for the investigation being reported | 6-7 |
| Objectives | 3 | State specific objectives, including any prespecified hypotheses | 7 |
| **Methods** | | | |
| Study design | 4 | Present key elements of study design early in the paper | 7 |
| Setting | 5 | Describe the setting, locations, and relevant dates, including periods of recruitment, exposure, follow-up, and data collection | 7 |
| Participants | 6 | (*a*) *Cohort study*—Give the eligibility criteria, and the sources and methods of selection of participants. Describe methods of follow-up<br>*Case-control study*—Give the eligibility criteria, and the sources and methods of case ascertainment and control selection. Give the rationale for the choice of cases and controls<br>*Cross-sectional study*—Give the eligibility criteria, and the sources and methods of selection of participants | 7-8 |
| | | (*b*) *Cohort study*—For matched studies, give matching criteria and number of exposed and unexposed<br>*Case-control study*—For matched studies, give matching criteria and the number of controls per case | N/A |
| Variables | 7 | Clearly define all outcomes, exposures, predictors, potential confounders, and effect modifiers. Give diagnostic criteria, if applicable | 8 |
| Data sources/ measurement | 8* | For each variable of interest, give sources of data and details of methods of assessment (measurement). Describe comparability of assessment methods if there is more than one group | *9-10* |
| Bias | 9 | Describe any efforts to address potential sources of bias | 7-11 |
| Study size | 10 | Explain how the study size was arrived at | 10 |
| Quantitative variables | 11 | Explain how quantitative variables were handled in the analyses. If applicable, describe which groupings were chosen and why | 8-10 |
| Statistical methods | 12 | (*a*) Describe all statistical methods, including those used to control for confounding | 10-11 |
| | | (*b*) Describe any methods used to examine subgroups and interactions | 10-11 |
| | | (*c*) Explain how missing data were addressed | 10-11 |
| | | (*d*) *Cohort study*—If applicable, explain how loss to follow-up was addressed<br>*Case-control study*—If applicable, explain how matching of cases and controls was addressed<br>*Cross-sectional study*—If applicable, describe analytical methods taking account of sampling strategy | 10-11 |

For peer review only



For peer review only - http://bmjopen.bmj.com/site/about/guidelines.xhtml

(*e*) Describe any sensitivity analyses

Continued on next page

For peer review only

| | | | |
|---|---|---|---|
| **Results** | | | |
| Participants | 13* | (a) Report numbers of individuals at each stage of study—eg numbers potentially eligible, examined for eligibility, confirmed eligible, included in the study, completing follow-up, and analysed | 11 |
| | | (b) Give reasons for non-participation at each stage | N/A |
| | | (c) Consider use of a flow diagram | Considered |
| Descriptive data | 14* | (a) Give characteristics of study participants (eg demographic, clinical, social) and information on exposures and potential confounders | 12-14 |
| | | (b) Indicate number of participants with missing data for each variable of interest | 12-15 |
| | | (c) *Cohort study*—Summarise follow-up time (eg, average and total amount) | N/A |
| Outcome data | 15* | *Cohort study*—Report numbers of outcome events or summary measures over time | |
| | | *Case-control study*—Report numbers in each exposure category, or summary measures of exposure | |
| | | *Cross-sectional study*—Report numbers of outcome events or summary measures | *11* |
| Main results | 16 | (*a*) Give unadjusted estimates and, if applicable, confounder-adjusted estimates and their precision (eg, 95% confidence interval). Make clear which confounders were adjusted for and why they were included | 11 |
| | | (*b*) Report category boundaries when continuous variables were categorized | 13-15 |
| | | (*c*) If relevant, consider translating estimates of relative risk into absolute risk for a meaningful time period | N/A |
| Other analyses | 17 | Report other analyses done—eg analyses of subgroups and interactions, and sensitivity analyses | 13-15 |
| **Discussion** | | | |
| Key results | 18 | Summarise key results with reference to study objectives | 16-18 |
| Limitations | 19 | Discuss limitations of the study, taking into account sources of potential bias or imprecision. Discuss both direction and magnitude of any potential bias | 18 |
| Interpretation | 20 | Give a cautious overall interpretation of results considering objectives, limitations, multiplicity of analyses, results from similar studies, and other relevant evidence | 16-18 |
| Generalisability | 21 | Discuss the generalisability (external validity) of the study results | 16-18 |
| **Other information** | | | |
| Funding | 22 | Give the source of funding and the role of the funders for the present study and, if applicable, for the original study on which the present article is based | 19 |

*Give information separately for cases and controls in case-control studies and, if applicable, for exposed and unexposed groups in cohort and cross-sectional studies.

**Note:** An Explanation and Elaboration article discusses each checklist item and gives methodological background and published examples of transparent reporting. The STROBE checklist is best used in conjunction with this article (freely available on the Web sites of PLoS Medicine at http://www.plosmedicine.org/, Annals of Internal Medicine at http://www.annals.org/, and Epidemiology at http://www.epidem.com/). Information on the STROBE Initiative is available at www.strobe-statement.org.

For peer review only

BMJ Open

# Who is engaging with lateral flow testing for COVID-19 in the UK? The COVID-19 Rapid Survey Of Adherence To Interventions And Responses (CORSAIR) study

| | |
|---|---|
| Journal: | *BMJ Open* |
| Manuscript ID | bmjopen-2021-058060.R1 |
| Article Type: | Original research |
| Date Submitted by the Author: | 06-Dec-2021 |
| Complete List of Authors: | Smith, Louise; Institute of Psychiatry Psychology and Neuroscience<br>Potts, Henry; University College London, Institute of Health Informatics<br>Amlot, Richard; UK Health Security Agency<br>Fear, Nicola; King's College London, Academic Centre for Defence Mental Health<br>Michie, Susan; University College London, Centre for Behaviour Change<br>Rubin, GJ; King's College London |
| <b>Primary Subject Heading</b>: | Public health |
| Secondary Subject Heading: | Health policy, Infectious diseases |
| Keywords: | COVID-19, Public health < INFECTIOUS DISEASES, PUBLIC HEALTH |
| | |

SCHOLARONE™
Manuscripts

*I, the Submitting Author has the right to grant and does grant on behalf of all authors of the Work (as defined in the below author licence), an exclusive licence and/or a non-exclusive licence for contributions from authors who are: i) UK Crown employees; ii) where BMJ has agreed a CC-BY licence shall apply, and/or iii) in accordance with the terms applicable for US Federal Government officers or employees acting as part of their official duties; on a worldwide, perpetual, irrevocable, royalty-free basis to BMJ Publishing Group Ltd ("BMJ") its licensees and where the relevant Journal is co-owned by BMJ to the co-owners of the Journal, to publish the Work in this journal and any other BMJ products and to exploit all rights, as set out in our licence.*

*The Submitting Author accepts and understands that any supply made under these terms is made by BMJ to the Submitting Author unless you are acting as an employee on behalf of your employer or a postgraduate student of an affiliated institution which is paying any applicable article publishing charge ("APC") for Open Access articles. Where the Submitting Author wishes to make the Work available on an Open Access basis (and intends to pay the relevant APC), the terms of reuse of such Open Access shall be governed by a Creative Commons licence – details of these licences and which Creative Commons licence will apply to this Work are set out in our licence referred to above.*

*Other than as permitted in any relevant BMJ Author's Self Archiving Policies, I confirm this Work has not been accepted for publication elsewhere, is not being considered for publication elsewhere and does not duplicate material already published. I confirm all authors consent to publication of this Work and authorise the granting of this licence.*

For peer review only

# Who is engaging with lateral flow testing for COVID-19 in the UK? The COVID-19 Rapid Survey of Adherence to Interventions and Responses (CORSAIR) study

Louise E Smith (0000-0002-1277-2564),[1,2] PhD, Henry WW Potts (0000-0002-6200-8804),[3] PhD, Richard Amlôt (0000-0003-3481-6588),[2,4] PhD, Nicola T Fear (0000-0002-5792-2925),[1,5] DPhil (Oxon), Susan Michie (0000-0003-0063-6378),[6] DPhil, G James Rubin (0000-0002-4440-0570),[1,2] PhD

1 King's College London, Institute of Psychiatry, Psychology and Neuroscience

2 NIHR Health Protection Research Unit in Emergency Preparedness and Response

3 University College London, Institute of Health Informatics

4 UK Health Security Agency, Behavioural Science and Insights Unit

5 King's Centre for Military Health Research and Academic Department of Military Mental Health

6 University College London, Centre for Behaviour Change

Corresponding author: Louise E Smith, Post-doctoral Researcher. Department of Psychological Medicine, King's College London, Weston Education Centre, Cutcombe Road, London, SE5 9RJ. Email: louise.e.smith@kcl.ac.uk. ORCID number: 0000-0002-1277-2564. Twitter handle: @louisesmith142

Henry WW Potts, Professor of Health Informatics. Institute of Health Informatics, 222 Euston Road, London, NW1 2DA.

Richard Amlôt, Head of Behavioural Science in the Behavioural Science and Insights Unit at UK Health Security Agency. Porton Down, Salisbury, Wiltshire, SP4 0JG.

Nicola T Fear, Professor of Epidemiology. Department of Psychological Medicine, King's College London, Weston Education Centre, Cutcombe Road, London, SE5 9RJ.



For peer review only - http://bmjopen.bmj.com/site/about/guidelines.xhtml

Susan Michie, Professor of Health Psychology. Centre for Behaviour Change, University College London,1-19 Torrington Place, London, WC1E 7HB.

G James Rubin, Assistant Director, NIHR Health Protection Research Unit in Emergency Preparedness and Response, King's College London, Weston Education Centre, Cutcombe Road, London, SE5 9RJ.

For peer review only



For peer review only - http://bmjopen.bmj.com/site/about/guidelines.xhtml

## ABSTRACT

Objectives: To investigate uptake of lateral flow testing, reporting of test results, and psychological, contextual, and socio-demographic factors associated with testing.

Design: Series of four fortnightly online cross-sectional surveys.

Setting: Data collected 19 April to 2 June 2021.

Participants: People living in England and Scotland, aged 18 years or over, excluding those who reported their most recent test was a polymerase chain reaction (PCR) test (n=6646, n≈1,600 per survey).

Main outcome measures: Having completed at least one lateral flow test (LFT) in the last seven days.

Results: We used binary logistic regressions to investigate factors associated with having taken at least one LFT. Increased uptake of testing was associated with being vaccinated (aORs=1.52 to 2.45, 95% CI 1.25 to 3.07, analysed separately by vaccine dose), employed (aOR=1.94, 95% CI 1.63 to 2.32), having been out to work in the last week (aOR=2.30, 95% CI 1.94 to 2.73), and working in a sector that adopted LFT early (aOR=2.54, 95% CI 2.14 to 3.02) . Uptake was higher in people who reported cardinal COVID-19 symptoms in the last week (aOR=1.89, 95% CI 1.34 to 2.66). People who had heard more about LFTs (aOR=2.28, 95% CI 2.06 to 2.51) and knew they were eligible to receive regular LFTs (aOR=2.98, 95% CI 2.35 to 3.78) were also more likely to have tested. Factors associated with not taking a test included agreeing that you do not need to test for COVID-19 unless you have come into contact with a case (aOR=0.51, 95% CI 0.47 to 0.55).

Conclusions: Uptake of lateral flow testing is low. Encouraging testing through workplaces and places of study is likely to increase uptake, although care should be taken not to pressurise employees and students. Increasing knowledge that everyone is eligible for regular asymptomatic testing and addressing common misconceptions may drive uptake.


Keywords: COVID-19, antigen testing, lateral flow testing, mass asymptomatic testing, uptake, predictors

Abstract word count: 300



For peer review only - http://bmjopen.bmj.com/site/about/guidelines.xhtml

## STRENGTHS AND LIMITATIONS OF THIS STUDY

- Series of large cross-sectional surveys investigating uptake, and factors associated with uptake, of lateral flow testing in the English and Scottish population.
- One of few studies investigating psychological, contextual, and socio-demographic factors associated with uptake of testing.
- Data were collected in the weeks immediately after the recommendation that all UK adults should take two rapid, lateral flow tests per week.
- Self-reported rates of having completed a lateral flow test in the last week were higher than estimated by official agencies.
- The behaviour and beliefs of people who have signed up to complete internet surveys may not be representative of those of the general population.

For peer review only



For peer review only - http://bmjopen.bmj.com/site/about/guidelines.xhtml

# INTRODUCTION

As the UK moves to continuous management of COVID-19 instead of disaster prevention, a variety of strategies are being used to slow the spread of infection. It is thought that transmission while asymptomatic accounts for 24% of COVID-19 transmission.(1) There is limited evidence of the effectiveness of mass asymptomatic testing programmes at slowing the spread of COVID-19 as most programmes, both in the UK and abroad, have been used in conjunction with other behavioural restrictions making it impossible to quantify the impact of mass testing alone.(2, 3) However, the effectiveness of any intervention will be limited if people do not engage with that behaviour.

Since 9 April 2021, everyone in the UK has been able to access free, regular, rapid lateral flow COVID-19 testing for use when asymptomatic.(4) At the time of writing, the English and Scottish Governments are recommending twice weekly lateral flow testing for all adults. Lateral flow tests (LFTs) for asymptomatic testing can be ordered online, collected from NHS pharmacies and are supplied through schools, colleges and nurseries; some universities and other employers also offer rapid testing.(5) Results of these tests should be reported through a UK Government website.(6) Mass asymptomatic testing was piloted in Liverpool, England between 6 November 2020 and 30 April 2021. Findings indicated that 57% of residents in Liverpool took at least one rapid LFT over the course of the pilot (~6 months), but uptake was substantially lower in more deprived areas (where infection rates were higher) and among non-white minoritized ethnic groups.(7, 8) Testing in the initial months was partly driven by the intense media campaign in Liverpool, and the novelty of the testing at the time. As well as asymptomatic testing being available, all those with cardinal COVID-19 symptoms (high temperature, a new, continuous cough, a loss or change to sense of smell or taste) have been eligible for a polymerase chain reaction (PCR) test since 18 May 2020.(9) There is considerable confusion among the public about the use of LFT and PCR tests when symptomatic.(10) Previous research indicates that uptake of testing when symptomatic is low.(11)

A range of factors may affect whether people engage with lateral flow testing. These can be categorised using the Capability, Opportunity, Motivation and Behaviour (COM-B) model.(12) Capability encompasses the psychological and physical capacity to engage in a behaviour. It includes, for example, knowledge as to what the appropriate behaviour is (e.g. knowing that you are eligible for regular testing) and when to enact it. Opportunity relates to



For peer review only - http://bmjopen.bmj.com/site/about/guidelines.xhtml

factors outside the person, for example testing being required by one's employer, or belonging to a group that was eligible for asymptomatic testing prior to the nationwide rollout (e.g. health and social care workers, teachers, students and people who work in transport such as hauliers) (13-16) and that, in turn, may be associated with socio-economic status or ethnicity. Motivation describes the psychological processes that energise or inhibit a behaviour and includes the perceived risk associated with a disease outbreak, which may in turn be linked to greater exposure to other people (e.g. during socialising),(17) believing that you have immunity against COVID-19,(18) believing that you could engage in a behaviour if you wanted to (self-efficacy), and perceiving the behaviour to be effective.

The aim of this study was to investigate rates of uptake of lateral flow testing and reporting of results in England and Scotland, and the psychological, contextual, and socio-demographic factors associated with testing.

# METHODS

## Design

From January 2020, BMG Research was conducting a series of nationally representative (UK) cross-sectional surveys (weekly or fortnightly) on behalf of the Department of Health and Social Care throughout the COVID-19 outbreak. We analysed these data as part of the CORSAIR study [the COVID-19 Rapid Survey of Adherence to Interventions and Responses study]).(11) For this study, we used data collected between 19 April and 2 June 2021 (rounds 48 to 51).

## Participants

Participants (n≈2,000 per survey) were eligible for the study if they were aged 16 years or over and lived in the UK and were recruited from two specialist research panel providers, Respondi (n=50,000) and Savanta (n=31,500). Quotas were applied based on age and gender (combined) to try to match these characteristics to those of the general UK population structure. Consent was implied by participants' completion of the survey, in line with industry standards. After completing the survey, participants were then unable to participate in the subsequent three surveys; thus, all participants included in this study were unique. Participants were reimbursed in points which could be redeemed in cash, gift vouchers or charitable donations (up to 70p).



For peer review only - http://bmjopen.bmj.com/site/about/guidelines.xhtml

We limited the sample to people living in England or Scotland as Wales and Northern Ireland are following a different testing schedule. We excluded those under 18 years of age as many would be eligible for asymptomatic testing under school testing regimes.

To investigate uptake of lateral flow testing, we excluded people who reported that their most recent test was a PCR test or they did not know what type their most recent test was and who reported that they had completed a PCR test or who did not know what type of test they completed after developing COVID-19 symptoms.

## Study materials

### Lateral flow testing

Participants were asked "when was the last time [they] had a test for coronavirus". Response options ranged from "within the last 24 hours" to "I've never had a coronavirus test".

People who reported having a COVID-19 test in the last week were asked a series of follow-up questions. These included how many times they had taken a COVID-19 test in the last seven days" (responses from "once" to "ten times or more"); how they received their most recent COVID-19 test (response options included receiving it from a care home, one's place of work, a school, further education college, or university, a hospital/clinical setting, having ordered it online, collecting a pack from a local test site or taking an assisted test at a local test site, or when travelling internationally); where they reported their test result, if at all (response options included the GOV.UK website, by phone with NHS Test and Trace, to one's employer, a school, further education college, or university, and to friends and family); what the result of their most recent test was (response options: "I tested positive", "I tested negative", "The test was void (inconclusive)", "I have not received my results yet"); and which type of test they had most recently taken (response options: "PCR test", "lateral flow test" or "don't know/unsure"). For this question, participants were given an explanation of each test ("PCR tests are sent to a lab for processing and results are sent to you usually by text or email. This includes home test kits which you need to mail in or drop off. Rapid lateral flow tests provide results within 30 minutes of taking the test (these might also be referred to as rapid antigen tests). Both tests involve swabbing the back of your nose and throat").

All participants were asked how much they had previously heard about "free, regular rapid testing for people even if they don't have coronavirus symptoms, which uses a technology called 'lateral flow testing'" on a four-point scale from "nothing at all" to "a great deal". They were also asked if "as far as [they knew, they were] eligible to receive rapid COVID-19



For peer review only - http://bmjopen.bmj.com/site/about/guidelines.xhtml

tests twice a week to check for coronavirus even if [they didn't] have symptoms (also known as lateral flow testing)?". Possible responses were "yes", "no" and "don't know".

## Contextual factors

All participants were asked if they had had any symptoms in the past seven days from a list of ten (new, continuous cough; high temperature / fever; runny nose; diarrhoea; nausea / feeling sick; vomiting; sneezing; loss of appetite; loss of sense of smell; loss of taste). In the final round of data collection, this question changed to ask if participants had developed any symptoms in the past ten days (response options remained identical).

We also identified people in groups who had been eligible for asymptomatic testing before it became available to everyone (people who indicated that they worked or volunteered in health or social care, education and childcare, or transport). Participants were identified as students if they specified that their employment status was "student / on a government training programme (Nation Traineeship/Modern Apprentice)".

Participants were asked how many times in the last seven days they had been out of their home to go to work and to meet up with friends or family that they did not live with (responses capped at 30). In the last round of data collection, this question was changed to ask how many times in the past seven days they had "left the house to go out to work (number of days)" and "met up with" friends or family they did not live with (responses capped at 30). We recoded the number of times people had been out for work into a binary variable (been out to work in the last week vs not).

## Psychological factors

Participants were asked how much, if at all, they agreed that: they were confident that lateral flow tests were accurate; regularly testing people without symptoms is an effective way to prevent COVID-19 transmission; you do not need to take a lateral flow test unless you have come into contact with a COVID-19 case; and that people who have been vaccinated do not need to be tested for COVID-19 regularly. All questions were asked on a scale from "strongly disagree" to "strongly agree".

Perceived risk of COVID-19 was measured by asking participants to what extent they thought COVID-19 posed a risk to "you personally" and "people in the UK" on a five-point scale from "no risk at all" to "major risk".

For peer review only



For peer review only - http://bmjopen.bmj.com/site/about/guidelines.xhtml

Socio-demographic factors

Participants were asked for their sex, age, employment status, socio-economic grade, highest educational or professional qualification, ethnicity, first language and COVID-19 vaccination status. Participants were also asked whether they had any dependent children in their household and whether they or a member of their household had a chronic illness. We computed a quadratic term for age. Region and index of multiple deprivation was derived from participants' postcode.(19)

Participants were also asked if they had had, or currently had, COVID-19. We recoded responses to give a binary variable (think have had COVID-19 ["I've definitely had it, and had it confirmed by a test", "I think I've probably had it"] vs think have not had COVID-19 ["I don't know whether I've had it or not", "I think I've probably not had it", "I've definitely not had it"]).

We measured financial hardship by asking participants to what extent in the past seven days they had been struggling to make ends meet, skipping meals they would usually have, and were finding their current living situation difficult (Cronbach's $\alpha$=.83).

## Ethics

This work was conducted as a service evaluation of the Department of Health and Social Care's public communications campaign and, following advice from King's College London Research Ethics Subcommittee, was exempt from ethical approval.

## Patient and public involvement

Lay members served on the advisory group for the project that developed our prototype survey material; this included three rounds of qualitative testing.(20) Due to the rapid nature of this research, the public was not involved in the further development of the materials during the COVID-19 pandemic.

## Power

We used a post-hoc power calculation to calculate power achieved in logistic regression analyses. With a sample size of 6646, we had 100% power to detect small odds ratios (OR=1.67) (21) at $\alpha$=0.003 (probability of taking an LFT in last week = 0.169).



For peer review only - http://bmjopen.bmj.com/site/about/guidelines.xhtml

## Analysis

We report uptake of lateral flow testing, how people received their most recent test, and out-of-home behaviour following a positive test result descriptively.

Contextual, psychological, and socio-demographic factors were identified *a priori*, based on theory (COM-B model).(12) We ran multivariable logistic regression analyses to investigate contextual, psychological, and socio-demographic factors associated with having completed at least one COVID-19 test in the last week, controlling for survey round, region, gender, age (raw and quadratic), presence of a dependent child in the household, having a chronic illness oneself, having a household member who has chronic illness, employment status, socio-economic grade, index of multiple deprivation quartile (2019),(19) highest educational or professional qualification, ethnicity, first language, having had COVID-19 before, vaccination status, and financial hardship. For these analyses, we excluded people who reported that they did not know when their last COVID-19 test was. As we hypothesised that peoples' beliefs about the necessity for regular asymptomatic testing of people who had been vaccinated for COVID-19 might differ based on vaccination status, we investigated associations between this belief and increased uptake of testing separately in those who reported having no, one, or two doses of a COVID-19 vaccine. We also hypothesised that participants who received a positive COVID-19 test or whose test result was inconclusive may perceive the risk of COVID-19 to themselves differently to those who had received a negative test. Therefore, we also investigated associations between perceived risk to oneself and increased uptake of testing excluding people whose most recent test result was positive or inconclusive.

Due to the large number of analyses (n=16) conducted on a single outcome, we used a Bonferroni correction and only report narratively results where $p<.003$. Tables give raw $p$-values.

We conducted a sensitivity analysis, running logistic regression analyses using index of multiple deprivation as a categorical variable.

# RESULTS

## Participant characteristics

Of participants included in analyses, 54% were female, with participants having a mean age of 49 years (Table 1).



For peer review only - http://bmjopen.bmj.com/site/about/guidelines.xhtml

Table 1. Participant characteristics

| Participant characteristic | Level | N | Percentage |
|---|---|---|---|
| Sex | Male | 3025 | 45.5 |
| | Female | 3600 | 54.2 |
| | Other / prefer not to say | 21 | 0.3 |
| Age | Range 18 to 91 years | Mean = 49.4 years | SD = 16.6 |
| Region | East Midlands | 539 | 8.1 |
| | East of England | 713 | 10.7 |
| | London | 815 | 12.3 |
| | North East | 312 | 4.7 |
| | North West | 815 | 12.3 |
| | Scotland | 685 | 10.3 |
| | South East | 924 | 13.9 |
| | South West | 604 | 9.1 |
| | West Midlands | 627 | 9.4 |
| | Yorkshire and the Humber | 612 | 9.2 |
| Ethnicity | White British | 5590 | 84.1 |
| | White other | 397 | 6.0 |
| | Mixed | 137 | 2.1 |
| | Asian / Asian British | 305 | 4.6 |
| | Black / Black British | 142 | 2.1 |
| | Arab / other | 34 | 0.5 |
| | Prefer not to say | 41 | 0.6 |
| Socio-economic grade | ABC1 | 4688 | 70.5 |
| | C2DE | 1812 | 27.3 |
| | Prefer not to say | 146 | 2.2 |
| Index of Multiple Deprivation | 1st quartile (least deprived) | 1365 | 20.5 |
| | 2nd quartile | 1652 | 24.9 |
| | 3rd quartile | 1791 | 26.9 |
| | 4th quartile (most deprived) | 1838 | 27.7 |

## Uptake of lateral flow testing

Since the introduction of guidance recommending twice weekly asymptomatic testing for all adults, 16.9% (95% CI 16.0% to 17.8%, n=1123/6646) of people reported that they had had a lateral flow COVID-19 test in the last week, excluding those whose most recent test was a PCR test (Table 2). Of these, 65.2% had completed two or more tests in the last week (11.0% total sample).

Table 2. Uptake of lateral flow testing

| When was the last time you had a test for coronavirus? We're interested in your most recent test, even if you didn't have symptoms [Total n=6646] | % (N) | *Asked to people who reported having a covid test in the last seven days.* And how many times have you taken a test for coronavirus in the last seven days? [Total n=1123] | % (N) |
|---|---|---|---|
| Within the last 24 hours | 4.1 (273) | Once | 34.3 (385) |
| 1-3 days ago | 7.4 (492) | Twice | 47.2 (530) |
| 4-7 days ago | 5.4 (358) | Three times | 9.4 (106) |
| One to two weeks ago | 7.1 (469) | Four to five times | 3.7 (42) |
| Two to four weeks ago | 6.9 (458) | Six to seven times | 2.8 (32) |



For peer review only - http://bmjopen.bmj.com/site/about/guidelines.xhtml

| | | | |
|---|---|---|---|
| One month to three months ago | 10.0 (666) | Eight to nine times | 0.7 (8) |
| Three months to six months ago | 7.8 (519) | Ten times or more | 1.2 (14) |
| More than six months ago | 6.3 (417) | | |
| I've never had a coronavirus test | 43.0 (2861) | | |
| Don't know | 2.0 (133) | Don't know | 0.5 (6) |

Most people reported the result of their most recent test to someone, with 64% reporting that they registered it with an official Government agency (Table 3).

Table 3. Where people register the results of their latest test

| *Asked to people who reported having a COVID-19 test in the last seven days.* How, if at all, did you report the result of your test? Tick all that apply [Total N=1123] | % (n) |
|---|---|
| I registered my result on GOV.UK | 50.0 (561) |
| I registered my result by phone with NHS Test & Trace | 17.0 (191) |
| I informed my employer | 15.4 (173) |
| I informed the school, nursery or further education college where I or a member of my family study | 6.9 (77) |
| I informed friends/family I was planning to meet after taking the test | 6.3 (71) |
| I informed friends/family I had recently met before taking the test | 5.0 (56) |
| I informed the university where I or a member of my family study | 2.5 (28) |
| Other | 2.0 (23) |
| I did not report the result to anyone | 15.8 (177) |
| Registered test result with GOV.UK or NHS Test & Trace | 64.1 (720) |
| Registered result with anyone (GOV.UK, NHS Test & Trace, one's employer, or the school, nursery, further education college, or university where the participant or a member of their family study) | 77.2 (867) |

## Associations with increased uptake of lateral flow testing

Increased uptake of lateral flow testing was associated with: being female, younger age, having a dependent child in your household, being employed, being vaccinated, having experienced COVID-19 symptoms in the last seven to ten days, being a student, having been out to work in the last week, working in a sector that adopted lateral flow testing early (health or social care, education and childcare, travel), having heard more about regular lateral flow testing, knowing that you are eligible for regular lateral flow testing, being confident that lateral flow tests are accurate, agreeing that regularly testing people without symptoms is an effective way to prevent the spread of COVID-19, and perceiving a greater risk of COVID-19 to people in the UK (Table 4).

Not having had a test was associated with not knowing that you were eligible for regular lateral flow testing, agreeing that you only need to take a lateral flow test if you have come into contact with somebody who has COVID-19, and that people who have been vaccinated do not need to be tested for coronavirus regularly (in people who reported at least one dose of



For peer review only - http://bmjopen.bmj.com/site/about/guidelines.xhtml

the vaccine; Table 4). There was significant variation by region, with Scotland showing lower uptake of lateral flow testing.

We ran a sensitivity analysis, conducting logistic regression analyses using index of multiple deprivation quartile as a categorical variable. This made minimal difference to the results.

For peer review only



For peer review only - http://bmjopen.bmj.com/site/about/guidelines.xhtml

Table 4. Factors associated with having completed at least one COVID-19 test in the last week. Bolding indicates findings significant at p<0.003.

| Factor | Level | Had not completed a COVID-19 test in the last week, n (%) [total n=5390] | Had completed a lateral flow COVID-19 test in the last week, n (%) [total n=1123] | Adjusted odds ratio (95% CI) for having completed a LFT † | p-value |
|---|---|---|---|---|---|
| Survey round | 19 to 21 April 2021 (round 48) | 1381 (84.6) | 252 (15.4) | Reference | - |
| | 4 to 5 May 2021 (round 49) | 1334 (81.3) | 306 (18.7) | 1.13 (0.93 to 1.38) | .22 |
| | 17 to 19 May 2021 (round 50) | 1326 (82.6) | 280 (17.4) | 1.04 (0.85 to 1.27) | .69 |
| | 1 to 2 June 2021 (round 51) | 1349 (82.6) | 285 (17.4) | 0.97 (0.79 to 1.19) | .78 |
| | Overall | - | - | $\chi^2(3)=2.8$ | .42 |
| Region | East Midlands | 443 (83.6) | 87 (16.4) | Reference | - |
| | East of England | 564 (80.3) | 138 (19.7) | 1.23 (0.90 to 1.68) | .19 |
| | London | 638 (80.7) | 153 (19.3) | 1.13 (0.83 to 1.55) | .43 |
| | North East | 251 (81.8) | 56 (18.2) | 1.22 (0.83 to 1.80) | .31 |
| | North West | 669 (84.2) | 126 (15.8) | 1.02 (0.75 to 1.40) | .89 |
| | Scotland | 608 (90.3) | 65 (9.7) | **0.53 (0.37 to 0.76)** | **.001** |
| | South East | 726 (80.3) | 178 (19.7) | 1.24 (0.92 to 1.67) | .15 |
| | South West | 504 (84.3) | 94 (15.7) | 1.04 (0.74 to 1.44) | .83 |
| | West Midlands | 489 (79.5) | 126 (20.5) | 1.35 (0.98 to 1.85) | .07 |
| | Yorkshire and the Humber | 498 (83.3) | 100 (16.7) | 1.08 (0.78 to 1.50) | .65 |
| | Overall | - | - | **$\chi^2(9)=35.6$** | **<.001** |
| Gender | Male | 2508 (85.0) | 442 (15.0) | Reference | - |
| | Female | 2868 (80.9) | 676 (19.1) | **1.32 (1.14 to 1.51)** | **<.001** |
| Age | Raw age | N=5390, M=50.6, SD=16.5 | N=1123, M=44.9, SD=15.9 | **0.76 (0.72 to 0.81)** | **<.001** |
| Age – quadratic $(\text{age-mean})^2$ | - | - | - | 1.0003 (1.0000 to 1.0006) | .06 |
| Dependent child in household | None | 3829 (85.3) | 660 (14.7) | Reference | - |
| | Child present | 1561 (77.1) | 463 (22.9) | **1.29 (1.10 to 1.51)** | **.001** |
| Has a chronic illness (oneself) | None | 3974 (82.2) | 819 (17.8) | Reference | - |
| | Present | 1478 (83.6) | 209 (16.4) | 1.18 (1.00 to 1.39) | .05 |
| Household member has chronic illness | None | 4482 (82.7) | 936 (17.3) | Reference | - |
| | Present | 790 (82.0) | 173 (18.0) | 1.04 (0.86 to 1.26) | .71 |
| Employment status | Not working | 2544 (88.5) | 330 (11.5) | Reference | - |
| | Working | 2783 (78.1) | 781 (21.9) | **1.94 (1.63 to 2.32)** | **<.001** |
| Socio-economic grade | ABC1 | 3844 (83.6) | 754 (16.4) | Reference | - |
| | C2DE | 1438 (80.5) | 348 (19.5) | 1.10 (0.94 to 1.29) | .21 |
| Index of multiple deprivation ‡ | 1st quartile (least deprived) | 1125 (83.7) | 219 (16.3) | 0.93 (0.87 to 0.99) | .02 |



For peer review only - http://bmjopen.bmj.com/site/about/guidelines.xhtml

| | | | | | |
|---|---|---|---|---|---|
| | 2nd quartile | 1319 (80.8) | 313 (19.2) | | |
| | 3rd quartile | 1439 (82.1) | 314 (17.9) | | |
| | 4th quartile (most deprived) | 1507 (84.5) | 277 (15.5) | | |
| Highest educational or professional qualification | GCSE/vocational/A-level/No formal qualifications | 3625 (82.9) | 749 (17.1) | Reference | - |
| | Degree or higher (Bachelors, Masters, PhD) | 1765 (82.5) | 374 (17.5) | 0.91 (0.78 to 1.07) | .24 |
| Ethnicity | White British | 4549 (82.7) | 954 (17.3) | Reference | - |
| | White other | 323 (83.9) | 62 (16.1) | 0.87 (0.61 to 1.25) | .45 |
| | Black and minority ethnicity | 484 (82.2) | 105 (17.8) | 0.83 (0.64 to 1.08) | .16 |
| | Overall | - | - | $\chi^2(2)$=2.1 | .34 |
| English as first language | No | 415 (83.2) | 84 (16.8) | Reference | - |
| | Yes | 4975 (82.7) | 1039 (17.3) | 1.19 (0.86 to 1.65) | .29 |
| Had COVID-19 before | Think not | 4600 (83.8) | 888 (16.2) | Reference | - |
| | Think yes | 790 (77.1) | 235 (22.9) | 1.23 (1.03 to 1.47) | .02 |
| Vaccination status | Not vaccinated | 1628 (82.9) | 336 (17.1) | Reference | - |
| | 1 dose | 1937 (83.2) | 392 (16.8) | **1.52 (1.25 to 1.86)** | **<.001** |
| | 2 doses | 1825 (82.2) | 395 (17.8) | **2.45 (1.96 to 3.07)** | **<.001** |
| | Overall | - | - | **$\chi^2(2)$=61.7** | **<.001** |
| Financial hardship | Range 3 (least) to 15 (most) | N=5311, M=7.3, SD=3.0 | N=1107, M=7.5, SD=3.0 | 0.99 (0.97 to 1.01) | .38 |
| COVID-19 symptoms in last week / ten days | No | 5258 (83.2) | 1061 (16.8) | Reference | - |
| | Yes | 132 (68.0) | 62 (32.0) | **1.89 (1.34 to 2.66)** | **<.001** |
| Being a student | No | 5189 (83.0) | 1061 (17.0) | Reference | - |
| | Yes | 138 (73.4) | 50 (26.6) | **2.65 (1.76 to 4.00)** | **<.001** |
| Been out to work in last week | No | 3702 (88.3) | 490 (11.7) | Reference | - |
| | Yes | 1688 (72.7) | 633 (27.3) | **2.30 (1.94 to 2.73)** | **<.001** |
| Number of times been out to meet people from another household socially | Range 0 to 30 | N=5390, M=0.9, SD=1.5, median=0 | N=1123, M=1.2, SD=1.6, median=1 | 1.05 (1.01 to 1.10) | .03 |
| Work in a sector that adopted LFT early | No | 4700 (86.3) | 744 (13.7) | Reference | - |
| | Yes | 690 (64.5) | 379 (35.5) | **2.54 (2.14 to 3.02)** | **<.001** |
| Amount heard about regular LFT | 4-point scale from “nothing at all” to “a great deal” | N=5253, M=2.8, SD=0.8 | N=1112, M=3.3, SD=0.7 | **2.28 (2.06 to 2.51)** | **<.001** |
| As far as you know, are you eligible to receive rapid COVID-19 tests twice a week to check for coronavirus even if you don't have symptoms (also known as lateral flow testing)? | No | 928 (90.5) | 97 (9.5) | Reference | - |
| | Don't know | 1718 (95.0) | 91 (5.0) | **0.59 (0.43 to 0.80)** | **.001** |
| | Yes | 2744 (74.6) | 935 (25.4) | **2.98 (2.35 to 3.78)** | **<.001** |
| | Overall | - | - | **$\chi^2(2)$=240.5** | **<.001** |
| I am confident that lateral flow tests are accurate | 5-point scale from “strongly disagree” to “strongly agree” | N=5131, M=3.3, SD=1.0 | N=1097, M=3.6, SD=0.9 | **1.40 (1.29 to 1.51)** | **<.001** |
| Regularly testing people without symptoms is an effective way to prevent the spread of coronavirus | 5-point scale from “strongly disagree” to “strongly agree” | N=5225, M=3.9, SD=0.9 | N=1115, M=4.3, SD=0.8 | **1.96 (1.77 to 2.16)** | **<.001** |



For peer review only - http://bmjopen.bmj.com/site/about/guidelines.xhtml

| | | | | | |
|---|---|---|---|---|---|
| I do not need to take a lateral flow test unless I have come into contact with somebody who has coronavirus | 5-point scale from "strongly disagree" to "strongly agree" | N=5061, M=2.6, SD=1.0 | N=1114, M=2.0, SD=1.1 | **0.51 (0.47 to 0.55)** | **<.001** |
| People who have been vaccinated do not need to be tested for coronavirus regularly | 5-point scale from "strongly disagree" to "strongly agree" | | | | |
| | In people who have not been vaccinated | N=1480, N=2.8, SD=1.1 | N=329, M=2.7, SD=1.1 | 0.90 (0.80 to 1.01) | .08 |
| | In people who have had one vaccine dose | N=1790, M=2.5, SD=1.0 | N=385, M=2.0, SD=1.1 | **0.54 (0.47 to 0.61)** | **<.001** |
| | In people who have had two vaccine doses | N=1644, M=2.6, SD=1.0 | N=392, M=2.0, SD=1.1 | **0.53 (0.47 to 0.60)** | **<.001** |
| Perceived risk of COVID-19 to self | 5-point scale from "no risk at all" to "major risk" | N=5343, M=3.0, SD=1.1 | N=1117, M=3.0, SD=1.1 | 1.04 (0.98 to 1.11) | .23 |
| | Excluding people who tested positive and whose test result was inconclusive | N=5343, M=3.0, SD=1.1 | N=1058, M=3.0, SD=1.1 | 1.03 (0.96 to 1.10) | .42 |
| Perceived risk of COVID-19 to people in the UK | 5-point scale from "no risk at all" to "major risk" | N=5326, M=3.5, SD=1.0 | N=1114, M=3.6, SD=0.9 | **1.13 (1.05 to 1.22)** | **.001** |

† Adjusting for survey round, region, gender, age (raw and quadratic), presence of a dependent child in the household, having a chronic illness oneself, having a household member who has chronic illness, employment status, socio-economic grade, index of multiple deprivation (continuous variable), highest educational or professional qualification, ethnicity, first language, having had COVID-19 before, vaccination status, and financial hardship.

‡ Treated as a continuous variable

Abbreviations: LFT=lateral flow test

For peer review only

# DISCUSSION

These data suggest that uptake of lateral flow testing is low, with approximately 17% of the sample reporting having a test in the last week. Of the total sample, only 11% report completing at least two LFTs in the last week, in line with Government recommendations.(4) This is slightly lower than another survey finding that 25% of English and Scottish adults reported taking regular COVID-19 tests (defined as at least once or twice a week; data collected 29 July 2021) although that was not in a nationally representative sample.(22) These data are not directly comparable with the Liverpool pilot, which reported uptake of testing over the complete duration of the pilot (almost 6 months), rather than uptake of people per week.(7, 8) In the first month of the pilot, 35% of people reported having taken up either LFT or PCR test.(23) Analyses of tests reported to the UK Government indicate that the number of LFTs registered had steadily declined from approximately 5.7 million LFTs (15 to 21 April 2021 (24)) to around 3.5 million (27 May and 2 June 2021).(25) This number includes tests taken by children and does not include tests that have not been officially registered on the UK Government website. However, our data would imply around 10 million LFTs should be reported each week by people aged over 17 years in England and Scotland alone, suggesting that our survey respondents may be more compliant (uptake of testing and / or reporting of testing) than the general population. This is corroborated by official figures estimating that approximately 21% of lateral flow tests are reported.(26) Our data indicate that 64% of participants' most recent tests had been registered with an official Government agency.

Factors related to employment or study were associated with uptake of lateral flow testing. People were more likely to report having a test in the last week if they were employed, had been out to work in the last week, and if they worked in a sector that recommended asymptomatic testing before the national guidance was implemented. Students were also more likely to report having tested in the last week. This could be because people were encouraged or compelled to take tests through their workplace, because they were more familiar with testing, or because they were more worried or perceived a greater risk of exposure to COVID-19 as they were going out to their place of work or study.(27) The current findings suggest that encouraging employees to take tests could drive uptake. However, this should be approached with caution. Qualitative research suggests that barriers to implementing testing in the workplace include perceived inaccuracy of LFTs and adding to



For peer review only - http://bmjopen.bmj.com/site/about/guidelines.xhtml

employee burden.(28) There are also ethical issues to consider in employers putting pressure on their employees, and there is a potential resulting lack of income if workers are unable to attend their place of work if they decline.(29) Mandating testing may result in negative attitudes towards testing becoming more entrenched.(30) Uptake could also be increased by making testing easier, e.g. at or very near to places of work or study, drop-in rather than appointment-based and with explicit paid time off for testing. From 4 October 2021, people must request a "collect code" online or by telephone in order to collect a packet of LFTs from pharmacies.(31) Previously this was not needed. How this has impacted uptake of LFTs is not yet clear, but it is likely that this change will make LFTs less accessible to some and has the potential to negatively impact uptake.

Uptake of lateral flow testing was higher in people who reported experiencing cardinal COVID-19 symptoms (high temperature, a new, continuous cough, a loss or change to sense of smell or taste) in the last week. UK Government recommendations state that people with cardinal COVID-19 symptoms should request a PCR, rather than rely on an LFT. It is clear that this requirement is not always being followed.(10) Research suggests that people with less severe SARS-CoV-2 manifest different symptoms at the start of infection.(32) Expanding the symptom set for eligibility for a PCR test is likely to increase testing burden on NHS Test and Trace. In England, all legal restrictions on social mixing were lifted on 19 July 2021.(33) Since this date, a 7-day average of over 20,000 new COVID-19 per day has been recorded,(34) with 527,077 (21 August 2021) to 1,175,617 (6 September 2021) Pillar 2 tests being conducted per day (data checked until 24 November 2021).(35) Official communications aiming to promote engagement with Government recommendations should emphasise that people with COVID-19 symptoms should request a PCR test, as should those who test positive using an LFT.

Socio-demographic factors associated with uptake of lateral flow testing included being younger, living with a dependent child, lower socio-economic grade, and thinking that you have had COVID-19 before. Previous research has found these factors to be consistently associated with non-adherence to behaviours that prevent the spread of COVID-19.(11, 18, 36) However, the association between increased lateral flow testing and lower age has also been found in other data, largely driven by those of working age being more likely to complete a test.(8, 22) One possible explanation may be that younger people are less likely to work from home.(29, 37) Therefore, these findings may be an artefact of people testing in relation to their work or study. Official figures of registered tests indicate that asymptomatic



For peer review only - http://bmjopen.bmj.com/site/about/guidelines.xhtml

testing in school aged children, who are “expected to test twice weekly” (16) under the supervision of their parents, is driving uptake, with numbers of tests conducted falling during the school holidays.(25) Parents may be likely to test themselves for COVID-19 while supervising their child’s test. We are unsure why those who think they have had COVID-19 before are more likely to engage with lateral flow testing. Increased uptake of testing was associated with having been vaccinated. This may reflect general adherence, with those being more likely to engage in preventive behaviours also being more likely to be vaccinated (itself a preventive behaviour).

These data indicate that people were more likely to engage in lateral flow testing if they had heard more about, and knew they were eligible for, regular LFTs. This is consistent with uptake of preventive behaviours in previous pandemics.(38) Similar to predictions from the Protection Motivation Theory,(39) perceiving testing to be more accurate and effective were associated with increased uptake.(40) Conversely, people who agreed that you only need to take a test if you have come into contact with a COVID-19 case, and that people who have been vaccinated do not need to be tested regularly were less likely to have taken a test in the last week. The latter belief was particularly strongly associated with low uptake in those who had been vaccinated. Taken together, these results suggest that media campaigns raising awareness that all adults are eligible for the mass asymptomatic testing programme are likely to increase uptake.

Testing alone cannot prevent transmission of disease. Only when used in conjunction with other behavioural interventions (e.g. staying at home, isolating) will testing prevent transmission. Due to its consistent use in combination with other interventions, it is difficult to determine the effectiveness of asymptomatic testing programmes alone.(2, 3) One study suggests that mass testing 5% of the UK population per week would lead to a 2% mean reduction in the reproductive rate of SARS-CoV-2.(41) However, this was before the implementation of vaccination. Test sensitivity and specificity, and prevalence of infection in the population, will affect the number of cases accurately identified and missed by tests.(42) Despite initial concern over the sensitivity of LFTs, especially in asymptomatic cases where people are testing themselves,(43) recent data suggest that LFTs have an absolute sensitivity of over 80% to detect individuals shedding SARS-CoV-2.(44)

We cannot be certain that the behaviour and beliefs of those that complete internet surveys are representative of those of the general population. This is reflected in the higher reporting



For peer review only - http://bmjopen.bmj.com/site/about/guidelines.xhtml

of lateral flow tests in our sample compared to that reported by official agencies. However, associations within the data are still likely to be informative.(45) Since data reflected self-reported behaviour, reports may be biased, and influenced by social desirability or poor recall. Given that we asked about behaviour in the past week, the influence of poor recall should be low. We also mitigated this by defining uptake as having completed one test in the last week, while Government guidelines suggest two COVID-19 tests per week should be completed. Although we have data on where participants received their lateral flow tests, we did not ask why they took their most recent tests and so cannot identify whether people are engaging in routine testing, or whether testing behaviour is driven by completing a test before socialising or attending work.

When used in tandem with self-isolation, testing can prevent the spread of COVID-19 by lowering the circulation of cases within the community. In the UK, recommendations state that people with cardinal symptoms should complete a PCR test alongside a twice-weekly asymptomatic mass testing programme. People who test positive using an LFT should then complete a PCR test. Mass testing programmes aim to identify cases in the population that may otherwise have been missed. However, their effectiveness is unclear. Our study suggests that uptake of lateral flow testing in the population is low. Interventions to prevent the spread of COVID-19 are unlikely to be effective if people do not engage with the behaviour. One reason for low uptake is that people do not know they are eligible for regular asymptomatic testing. Work- and study-related factors were associated with uptake of lateral flow testing. Encouragement of employees and students, especially those attending their place of work or study, to engage in asymptomatic testing may increase uptake. However, employers and educational institutions should exercise caution so as not to place undue pressure on employees and students to test. People with symptoms were more likely to have completed a test in the past week. Consideration should be given to how best to optimise testing in the UK (PCR and LFT), taking into account the full range of symptoms displayed early in SARS-CoV-2 infection, transmission when asymptomatic, financial cost of testing, and burden on the testing system. Communications aiming to promote engagement with Government recommended testing should highlight that people with cardinal COVID-19 symptoms should request a PCR test rather than take an LFT.



For peer review only - http://bmjopen.bmj.com/site/about/guidelines.xhtml

## AUTHOR CONTRIBUTION STATEMENT

All authors conceptualised the study and contributed to survey materials. LS completed analyses with guidance from HWWP and GJR. LS wrote the first draft of the manuscript. HWWP, RA, NTF, SM and GJR contributed to subsequent drafts of the manuscript. LS, HWWP, RA, NTF, SM and GJR approved the final manuscript. GJR is guarantor. The corresponding author attests that all listed authors meet authorship criteria and that no others meeting the criteria have been omitted.

## FUNDING SOURCES

This work was funded by the National Institute for Health Research (NIHR) Health Services and Delivery Research programme (NIHR project reference number (11/46/21)). Surveys were commissioned and funded by Department of Health and Social Care (DHSC), with the authors providing advice on the question design and selection. LS, RA and GJR are supported by the National Institute for Health Research Health Protection Research Unit (NIHR HPRU) in Emergency Preparedness and Response, a partnership between the UK Health Security Agency, King's College London and the University of East Anglia. RA is also supported by the NIHR HPRU in Behavioural Science and Evaluation, a partnership between the UK Health Security Agency and the University of Bristol. HWWP has received funding from Public Health England and NHS England. NTF is part funded by a grant from the UK Ministry of Defence. The views expressed are those of the authors and not necessarily those of the NIHR, UK Health Security Agency, the Department of Health and Social Care or the Ministry of Defence. The Department of Health and Social Care funded data collection (no grant number).

## COMPETING INTERESTS DECLARATION

All authors have completed the ICMJE uniform disclosure form at www.icmje.org/coi_disclosure.pdf and declare: all authors had financial support from NIHR for the submitted work; RA is an employee of the UK Health Security Agency; HWWP received additional salary support from Public Health England and NHS England; HWWP receives consultancy fees to his employer from Ipsos MORI and has a PhD student who works at and has fees paid by Astra Zeneca; no other financial relationships with any organisations that might have an interest in the submitted work in the previous three years; no other relationships or activities that could appear to have influenced the submitted work. NTF



For peer review only - http://bmjopen.bmj.com/site/about/guidelines.xhtml

is a participant of an independent group advising NHS Digital on the release of patient data. All authors are participants of the UK's Scientific Advisory Group for Emergencies or its subgroups.

## ETHICAL APPROVAL

This work was conducted as part of service evaluation of the marketing and communications run by the Department of Health and Social Care, and so did not require ethical approval.

## DATA SHARING STATEMENT

No additional data are available from the authors.

## TRANSPARENCY DECLARATION

The authors affirm that the manuscript is an honest, accurate, and transparent account of the study being reported; that no important aspects of the study have been omitted; and that any discrepancies from the study as originally planned have been explained.

Surveys were commissioned and funded by DHSC, with the authors providing advice on the question design and selection. DHSC had no role in analysis, decision to publish, or preparation of the manuscript. Preliminary results were made available to DHSC.

## DISSEMINATION DECLARATION

Dissemination of survey results to participants is not possible due to the anonymous nature of data collection.

## LICENSE

A CC BY license is required. The Corresponding Author has the right to grant on behalf of all authors and does grant on behalf of all authors, a worldwide licence (the BMJ license) to the Publishers and its licensees in perpetuity, in all forms, formats and media (whether known now or created in the future), to i) publish, reproduce, distribute, display and store the Contribution, ii) translate the Contribution into other languages, create adaptations, reprints, include within collections and create summaries, extracts and/or, abstracts of the Contribution and convert or allow conversion into any format including without limitation audio, iii) create any other derivative work(s) based in whole or part on the on the Contribution, iv) to exploit all subsidiary rights to exploit all subsidiary rights that currently exist or as may exist in the



For peer review only - http://bmjopen.bmj.com/site/about/guidelines.xhtml

future in the Contribution, v) the inclusion of electronic links from the Contribution to third party material where-ever it may be located; and, vi) licence any third party to do any or all of the above. All research articles will be made available on an open access basis (with authors being asked to pay an open access fee—see copyright, open access, and permission to reuse). The terms of such open access shall be governed by a Creative Commons licence—details as to which Creative Commons licence will apply to the research article are set out in our worldwide licence referred to above.

For peer review only



For peer review only - http://bmjopen.bmj.com/site/about/guidelines.xhtml

# REFERENCES

1. Johansson MA, Quandelacy TM, Kada S, Prasad PV, Steele M, Brooks JT, et al. SARS-CoV-2 Transmission From People Without COVID-19 Symptoms. *JAMA Netw Open.* 2021;4(1):e2035057.

2. Wise J. Covid-19: Concerns persist about purpose, ethics, and effect of rapid testing in Liverpool. *BMJ.* 2020;371:m4690.

3. Pavelka M, Van-Zandvoort K, Abbott S, Sherratt K, Majdan M, group CC-w, et al. The impact of population-wide rapid antigen testing on SARS-CoV-2 prevalence in Slovakia. *Science*. 2021;372(6542):635-41.

4. Department of Health and Social Care. New campaign urges public to get tested twice a week. 2021. Available from: https://www.gov.uk/government/news/new-campaign-urges-public-to-get-tested-twice-a-week.

5. NHS. Regular rapid lateral flow coronavirus (COVID-19) tests. 2021. Available from: https://www.nhs.uk/conditions/coronavirus-covid-19/testing/regular-rapid-coronavirus-tests-if-you-do-not-have-symptoms/.

6. GOV.UK. Report a COVID-19 rapid lateral flow test result. 2021.Available from: https://www.gov.uk/report-covid19-result.

7. University of Liverpool, NHS Test and Trace, Joint Biosecurity Centre, Public Health England, Statistics OfN. Liverpool Covid-SMART Community Testing Pilot; Evaluation Report. University of Liverpool. 2021. Available from: https://www.liverpool.ac.uk/media/livacuk/research/Mass,testing,evaluation.pdf.

8. Green MA, Garcia-Finana M, Barr B, Burnside G, Cheyne CP, Hughes D, et al. Evaluating social and spatial inequalities of large scale rapid lateral flow SARS-CoV-2 antigen testing in COVID-19 management: An observational study of Liverpool, UK (November 2020 to January 2021). *Lancet Reg Health Eur.* 2021;6:100107.

9. Everyone in the United Kingdom with symptoms now eligible for coronavirus tests [press release]. 2020. Available from: https://www.gov.uk/government/news/everyone-in-the-united-kingdom-with-symptoms-now-eligible-for-coronavirus-tests.

10. Smith LE, Potts HWW, Amlôt R, Fear NT, Michie S, Rubin GJ. Do members of the public think they should use lateral flow tests or PCR tests when they have COVID-19-like

For peer review only

symptoms? The COVID-19 Rapid Survey of Adherence to Interventions and Responses [CORSAIR] study. *Public Health.* 2021.

11. Smith LE, Potts HWW, Amlôt R, Fear NT, Michie S, Rubin GJ. Adherence to the test, trace, and isolate system in the UK: results from 37 nationally representative surveys. *BMJ.* 2021;372:n608.

12. Michie S, van Stralen MM, West R. The behaviour change wheel: a new method for characterising and designing behaviour change interventions. *Implement Sci.* 2011;6:42.

13. Chris Whitty, Stephen Powis. Asymptomatic NHS Staff Testing. 2020. Available from: https://cdn.ps.emap.com/wp-content/uploads/sites/3/2020/11/Prof-Chris-Whitty-Prof-Stephen-Powis-to-Chair-Asymptomatic-testing-of-NHS-Staff-09.11.20.pdf-FOR-WEBSITE.pdf.

14. Department for Transport. Get a coronavirus (COVID-19) test if you're an HGV or van driver. 2021. Available from: https://www.gov.uk/guidance/get-a-coronavirus-covid-19-test-if-youre-an-hgv-or-van-driver#history.

15. NHS Test and Trace. COVID-19 National Testing Programme: Schools & Colleges handbook. 2020. Available from: https://schoolsweek.co.uk/wp-content/uploads/2020/12/Schools_Colleges_Testing-Handbook_version-3.3-Copy.pdf.

16. Department for Education. Coronavirus (COVID-19) asymptomatic testing in schools and colleges. 2021. Available from: https://www.gov.uk/government/publications/coronavirus-covid-19-asymptomatic-testing-in-schools-and-colleges/coronavirus-covid-19-asymptomatic-testing-in-schools-and-colleges.

17. Dryhurst S, Schneider CR, Kerr J, Freeman ALJ, Recchia G, van der Bles AM, et al. Risk perceptions of COVID-19 around the world. *J Risk Res.* 2020:1-13.

18. Smith LE, Mottershaw AL, Egan M, Waller J, Marteau TM, Rubin GJ. The impact of believing you have had COVID-19 on self-reported behaviour: Cross-sectional survey. *PLoS One.* 2020;15(11):e0240399.

19. Ministry of Housing Communities and Local Government. The English Indices of Deprivation 2019 (IoD2019). 2019. Available from: https://assets.publishing.service.gov.uk/government/uploads/system/uploads/attachment_data/file/835115/IoD2019_Statistical_Release.pdf.



For peer review only - http://bmjopen.bmj.com/site/about/guidelines.xhtml

20. Rubin GJ, Bakhshi S, Amlot R, Fear N, Potts HWW, Michie S. The design of a survey questionnaire to measure perceptions and behaviour during an influenza pandemic: the Flu TElephone Survey Template (FluTEST). *Health Services and Delivery Research.* 2014;2(41).

21. Chen HN, Cohen P, Chen S. How Big is a Big Odds Ratio? Interpreting the Magnitudes of Odds Ratios in Epidemiological Studies. *Communications in Statistics - Simulation and Computation.* 2010;39(4):860-4.

22. YouGov. At the current time, are you or are you not taking regular Covid-19 tests (at least once or twice a week)? 2021. Available from: https://yougov.co.uk/topics/health/survey-results/daily/2021/07/29/f6d26/2.

23. Department of Health and Social Care, NHS Test and Trace, British Army, Public Health england, Liverpool City Council, University of Liverpool, et al. Liverpool Covid-SMART (Systematic Meaningful Asymptomatic Repeated Testing) Pilot; Addressing SARS-CoV-2 transmission and harms from Covid-19 restrictions, as one system. 2020 Available from: https://assets.publishing.service.gov.uk/government/uploads/system/uploads/attachment_data/file/950695/s0958-liverpool-covid-smart-evaluation.pdf.

24. Department of Health and Social Care. Weekly statistics for NHS Test and Trace (England) and coronavirus testing (UK): 15 April to 21 April 2021. 2021. Available from: https://assets.publishing.service.gov.uk/government/uploads/system/uploads/attachment_data/file/982248/Test_and_Trace_Week47.pdf.

25. Department of Health and Social Care. Weekly statistics for rapid asymptomatic testing in England: 27 May to 2 June 2021. 2021. Available from: https://www.gov.uk/government/publications/weekly-statistics-for-nhs-test-and-trace-england-27-may-to-2-june-2021/weekly-statistics-for-rapid-asymptomatic-testing-in-england-27-may-to-2-june-2021#lfd-tests-conducted-england1.

26. Department of Health and Social Care. Management information on lateral flow device (LFD) test registration rates: 22 July 2021. 2021. Available from: https://www.gov.uk/government/publications/management-information-on-lfd-test-registration-rates-22-july-2021/management-information-on-lateral-flow-device-lfd-test-registration-rates-22-july-2021#estimated-proportion-of-lfd-tests-dispatched-which-have-been-registered.



For peer review only - http://bmjopen.bmj.com/site/about/guidelines.xhtml

27. Kim Parker, Juliana Menasce Horowitz, Minkin R. How the Coronavirus Outbreak Has – and Hasn't – Changed the Way Americans Work. 2020. Available from: https://www.pewresearch.org/social-trends/2020/12/09/how-the-coronavirus-outbreak-has-and-hasnt-changed-the-way-americans-work/.

28. Kierkegaard P, Micocci M, McLister A, Tulloch JSP, Parvulescu P, Gordon AL, et al. Implementing lateral flow devices in long-term care facilities: experiences from the Liverpool COVID-19 community testing pilot in care homes- a qualitative study. *BMC Health Serv Res.* 2021;21(1):1153.

29. Michie S, Potts HWW, West R, Amlôt R, Smith LE, Fear NT, et al. Factors associated with nonessential workplace attendance during the Covid-19 pandemic in the UK in early 2021: evidence from cross-sectional surveys. *Public Health.* 2021.

30. Smith LE, Hodson A, Rubin GJ. Parental attitudes towards mandatory vaccination; a systematic review. *Vaccine*. 2021;39(30):4046-53.

31. Pharmaceutical Services Negotiating Committee. C-19 lateral flow device distribution service. 2021. Available from: https://psnc.org.uk/services-commissioning/advanced-services/c-19-lateral-flow-device-distribution-service/.

32. Sudre CH, Lee KA, Lochlainn MN, Varsavsky T, Murray B, Graham MS, et al. Symptom clusters in COVID-19: A potential clinical prediction tool from the COVID Symptom Study app. *Sci Adv.* 2021;7(12).

33. Boris Johnson. Prime Minister confirms move to Step 4 [press release]. 12 July 2021. 2021. Available from: https://www.gov.uk/government/news/prime-minister-confirms-move-to-step-4.

34. GOV.UK. Cases in England. 2021. Available from [updated 24 November 2021]: https://coronavirus.data.gov.uk/details/cases?areaType=nation&areaName=England.

35. GOV.UK. Testing in England. 2021. Available from [updated 24 November 2021]: https://coronavirus.data.gov.uk/details/testing?areaType=nation&areaName=England.

36. Wright L, Steptoe A, Fancourt D. Patterns of compliance with COVID-19 preventive behaviours: a latent class analysis of 20,000 UK adults. *J Epidemiology Community Health.* 2021;0:1-7.



For peer review only - http://bmjopen.bmj.com/site/about/guidelines.xhtml

37. Office for National Statistics. Coronavirus and homeworking in the UK: April 2020. 2020. Available from: https://www.ons.gov.uk/employmentandlabourmarket/peopleinwork/employmentandemployeetypes/bulletins/coronavirusandhomeworkingintheuk/april2020#homeworking-by-occupation.

38. Rubin GJ, Potts HWW, Michie S. The impact of communications about swine flu (influenza A H1N1v) on public responses to the outbreak: results from 36 national telephone surveys in the UK. *Health Technol Asses.* 2010;14(34):183-266.

39. Rogers RW, Prentice-Dunn S. Protection motivation theory. In: Gochman DS, editor. Handbook of health behavior research 1: Personal and social determinants: Plenum Press; 1997.

40. van Loenhout JAF, Vanderplanken K, Scheen B, Van den Broucke S, Aujoulat I. Determinants of adherence to COVID-19 measures among the Belgian population: an application of the protection motivation theory. *Archives of Public Health.* 2021;79(1):74.

41. Kucharski AJ, Klepac P, Conlan AJK, Kissler SM, Tang ML, Fry H, et al. Effectiveness of isolation, testing, contact tracing, and physical distancing on reducing transmission of SARS-CoV-2 in different settings: a mathematical modelling study. *Lancet Infect Dis.* 2020;20(10):1151-60.

42. Mercer TR, Salit M. Testing at scale during the COVID-19 pandemic. *Nature Reviews Genetics.* 2021;22(7):415-26.

43. Dinnes J, Deeks JJ, Berhane S, Taylor M, Adriano A, Davenport C, et al. Rapid, point-of-care antigen and molecular-based tests for diagnosis of SARS-CoV-2 infection. *Cochrane Database Syst Rev.* 2021;3:CD013705.

44. Petersen I, Crozier A, Buchan I, Mina MJ, Bartlett JW. Recalibrating SARS-CoV-2 Antigen Rapid Lateral Flow Test Relative Sensitivity from Validation Studies to Absolute Sensitivity for Indicating Individuals Shedding Transmissible Virus. *Clin Epidemiol.* 2021;13:935-40.

45. Kohler U. Possible Uses of Nonprobability Sampling for the Social Sciences. *Survey Methods: Insights from the Field*. 2019. Available from: https://surveyinsights.org/?p=10981.



For peer review only - http://bmjopen.bmj.com/site/about/guidelines.xhtml

STROBE Statement—checklist of items that should be included in reports of observational studies

| | Item No | Recommendation | Page No |
|---|---|---|---|
| **Title and abstract** | 1 | (*a*) Indicate the study's design with a commonly used term in the title or the abstract | 1, 3 |
| | | (*b*) Provide in the abstract an informative and balanced summary of what was done and what was found | 3 |
| **Introduction** | | | |
| Background/rationale | 2 | Explain the scientific background and rationale for the investigation being reported | 5-6 |
| Objectives | 3 | State specific objectives, including any prespecified hypotheses | 6 |
| **Methods** | | | |
| Study design | 4 | Present key elements of study design early in the paper | 6 |
| Setting | 5 | Describe the setting, locations, and relevant dates, including periods of recruitment, exposure, follow-up, and data collection | 6 |
| Participants | 6 | (*a*) *Cohort study*—Give the eligibility criteria, and the sources and methods of selection of participants. Describe methods of follow-up<br>*Case-control study*—Give the eligibility criteria, and the sources and methods of case ascertainment and control selection. Give the rationale for the choice of cases and controls<br>*Cross-sectional study*—Give the eligibility criteria, and the sources and methods of selection of participants | 6-7 |
| | | (*b*) *Cohort study*—For matched studies, give matching criteria and number of exposed and unexposed<br>*Case-control study*—For matched studies, give matching criteria and the number of controls per case | N/A |
| Variables | 7 | Clearly define all outcomes, exposures, predictors, potential confounders, and effect modifiers. Give diagnostic criteria, if applicable | 7-8 |
| Data sources/ measurement | 8* | For each variable of interest, give sources of data and details of methods of assessment (measurement). Describe comparability of assessment methods if there is more than one group | *8-9* |
| Bias | 9 | Describe any efforts to address potential sources of bias | 6-10 |
| Study size | 10 | Explain how the study size was arrived at | 6-7, 9-10 |
| Quantitative variables | 11 | Explain how quantitative variables were handled in the analyses. If applicable, describe which groupings were chosen and why | 6-10 |
| Statistical methods | 12 | (*a*) Describe all statistical methods, including those used to control for confounding | 10 |
| | | (*b*) Describe any methods used to examine subgroups and interactions | 10 |
| | | (*c*) Explain how missing data were addressed | 10 |
| | | (*d*) *Cohort study*—If applicable, explain how loss to follow-up was addressed<br>*Case-control study*—If applicable, explain how matching of cases and controls was addressed<br>*Cross-sectional study*—If applicable, describe analytical methods taking account of sampling strategy | N/A |
| | | (*e*) Describe any sensitivity analyses | 10 |


For peer review only - http://bmjopen.bmj.com/site/about/guidelines.xhtml
For peer review only

Continued on next page

For peer review only



For peer review only - http://bmjopen.bmj.com/site/about/guidelines.xhtml

| | | | |
|---|---|---|---|
| **Results** | | | |
| Participants | 13* | (a) Report numbers of individuals at each stage of study—eg numbers potentially eligible, examined for eligibility, confirmed eligible, included in the study, completing follow-up, and analysed | 10-12 |
| | | (b) Give reasons for non-participation at each stage | N/A |
| | | (c) Consider use of a flow diagram | Considered |
| Descriptive data | 14* | (a) Give characteristics of study participants (eg demographic, clinical, social) and information on exposures and potential confounders | 10-16 |
| | | (b) Indicate number of participants with missing data for each variable of interest | 11-16 |
| | | (c) *Cohort study*—Summarise follow-up time (eg, average and total amount) | N/A |
| Outcome data | 15* | *Cohort study*—Report numbers of outcome events or summary measures over time | |
| | | *Case-control study*—Report numbers in each exposure category, or summary measures of exposure | |
| | | *Cross-sectional study*—Report numbers of outcome events or summary measures | *11-16* |
| Main results | 16 | (*a*) Give unadjusted estimates and, if applicable, confounder-adjusted estimates and their precision (eg, 95% confidence interval). Make clear which confounders were adjusted for and why they were included | 11-16 |
| | | (*b*) Report category boundaries when continuous variables were categorized | 12-16 |
| | | (*c*) If relevant, consider translating estimates of relative risk into absolute risk for a meaningful time period | N/A |
| Other analyses | 17 | Report other analyses done—eg analyses of subgroups and interactions, and sensitivity analyses | 12-16 |
| **Discussion** | | | |
| Key results | 18 | Summarise key results with reference to study objectives | 17-20 |
| Limitations | 19 | Discuss limitations of the study, taking into account sources of potential bias or imprecision. Discuss both direction and magnitude of any potential bias | 19-20 |
| Interpretation | 20 | Give a cautious overall interpretation of results considering objectives, limitations, multiplicity of analyses, results from similar studies, and other relevant evidence | 17-20 |
| Generalisability | 21 | Discuss the generalisability (external validity) of the study results | 17-20 |
| **Other information** | | | |
| Funding | 22 | Give the source of funding and the role of the funders for the present study and, if applicable, for the original study on which the present article is based | 21-22 |

*Give information separately for cases and controls in case-control studies and, if applicable, for exposed and unexposed groups in cohort and cross-sectional studies.

**Note:** An Explanation and Elaboration article discusses each checklist item and gives methodological background and published examples of transparent reporting. The STROBE checklist is best used in conjunction with this article (freely available on the Web sites of PLoS Medicine at http://www.plosmedicine.org/, Annals of Internal Medicine at http://www.annals.org/, and Epidemiology at http://www.epidem.com/). Information on the STROBE Initiative is available at www.strobe-statement.org.

For peer review only
